# Robust Analysis of PM_2.5_ Concentration Measurements in the Ecuadorian Park La Carolina

**DOI:** 10.3390/s19214648

**Published:** 2019-10-25

**Authors:** Wilmar Hernandez, Alfredo Mendez, Angela Maria Diaz-Marquez, Rasa Zalakeviciute

**Affiliations:** 1 Facultad de Ingeniería y Ciencias Aplicadas, Universidad de Las Américas, Quito 170125, Ecuador; 2 Departamento de Matemática Aplicada a las Tecnologías de la Información y las Comunicaciones, ETS de Ingeniería y Sistemas de Telecomunicación, Universidad Politécnica de Madrid, 28040 Madrid, Spain; alfredo.mendez@upm.es; 3 Grupo Dinámicas + Lugar, Medio y Sociedad (D+LMS), Universidad de Las Américas, Quito 170125, Ecuador; angela.diaz@udla.edu.ec; 4 Grupo de Biodiversidad, Medio Ambiente y Salud (BIOMAS), Universidad de Las Américas, Quito 170125, Ecuador; rasa.zalakeviciute@udla.edu.ec

**Keywords:** PM_2.5_ concentration measurements, robust location estimation, robust scale estimation

## Abstract

In this article, a robust statistical analysis of particulate matter (PM_2.5_) concentration measurements is carried out. Here, the region chosen for the study was the urban park La Carolina, which is one of the most important in Quito, Ecuador, and is located in the financial center of the city. This park is surrounded by avenues with high traffic, in which shopping centers, businesses, entertainment venues, and homes, among other things, can be found. Therefore, it is important to study air pollution in the region where this urban park is located, in order to contribute to the improvement of the quality of life in the area. The preliminary study presented in this article was focused on the robust estimation of both the central tendency and the dispersion of the PM_2.5_ concentration measurements carried out in the park and some surrounding streets. To this end, the following estimators were used: (i) for robust location estimation: α-trimmed mean, trimean, and median estimators; and (ii) for robust scale estimation: median absolute deviation, semi interquartile range, biweight midvariance, and estimators based on a subrange. In addition, nonparametric confidence intervals were established, and air pollution levels due to PM_2.5_ concentrations were classified according to categories established by the Quito Air Quality Index. According to these categories, the results of the analysis showed that neither the streets that border the park nor the park itself are at the Alert level. Finally, it can be said that La Carolina Park is fulfilling its function as an air pollution filter.

## 1. Introduction

Air pollution is a serious problem [1]. Many people die every year due to problems related to air pollution. Therefore, in order to reduce this type of pollution in an efficient way, it is important to create citizen awareness about it and take care of green areas and trees, build more urban parks, avoid the use of aerosols, and improve the transportation system, among other things.

Living in the city implies that citizens have to face the problem of air pollution very often. Therefore, it is important to design measurement and control systems that can eliminate or mitigate the problems that air pollution is causing to humans and nature. The smart city concept takes this idea into account, and one of the most harmful pollutants for humans is PM_2.5_ (fine particulate matter with diameter smaller than 2.5 µm [2].

In [3], a method to perform the forecasting of PM_2.5_ concentrations in Taiwan was proposed. This method used the moving average technique to perform the forecast, and the authors considered that there was no seasonality or trend when analyzing short-term PM_2.5_ concentrations. The data used in [3] were obtained from PM_2.5_ sensing devices that were deployed all over Taiwan, and the forecast model shown in [3] used exponential smoothing with a drift to perform the prediction of PM_2.5_.

A low-cost air quality station designed to monitor environmental parameters and atmospheric pollutants was presented in [4]. Furthermore, in [4], the following variables were measured: air temperature, relative humidity, CO (carbon monoxide), CO_2_ (carbon dioxide), NO_2_ (nitrogen dioxide), O_3_ (ozone), VOC (volatile organic compound), PM_2.5_, and PM_10_ (particulate matter with diameter smaller than 10 µm); and the PM_2.5_ and PM_10_ sensors were calibrated. For the calibration process, linear regression, robust linear regression, and non-linear regression were used. In addition, the *M*-estimation method was used to reduce the influence of outliers on least squares fitting for robust linear regression.

Key components of a portable air pollution monitoring system based on low-cost sensors were introduced in [5]. In addition, in [5], the on-field testing of the sensing unit was described and roadside measurements from a mobile laboratory equipped with a calibrated instrument were used. Furthermore, in [5], a case study was presented in order to demonstrate the application by mounting the low-cost air pollution monitoring system on an electric bike. The experiment was carried out in the city of Mons, Belgium, and the results of the PM_2.5_ concentrations measurements were compared with measurements available from the local monitoring station. According to the authors of [5], that research paper was aimed at presenting a low-cost mobile system to map particulate matter concentration levels generated by the daily activity of the human being in the area under study.

A study aimed at evaluating the precision, accuracy, practicality, and potential uses of a PM_2.5_ miniaturized monitor was performed in [6]. Miniaturized monitors (AirBeam, HabitatMap) were compared with real-time particulate matter monitors of PM_2.5_ and a gravimetric reference method for PM_2.5_. The PM_2.5_ concentration measurements were carried out in Como, Italy. In [6], it was made clear that in the scientific literature, there are few articles aimed at performing the evaluation or comparison of miniaturized monitors. In addition, in [6], Shinyei PPD60PV sensors (AB) were chosen among others because of their practicability. Furthermore, several results of ABs evaluations that are reported in some scientific articles are also shown [6].

In order to carry out the statistical analysis and data treatment shown in [6], all data (except meteorological data averaged for a one-hour period) below the first percentile and above the 99th percentile were truncated. Also, a significance level of 5% was chosen for all tests. In addition, descriptive statistics were estimated for PM_2.5_ concentration measurements, and the following tests were used: (1) precision evaluation (in accordance with [7], the evaluation of the uncertainty between co-located miniaturized monitors by means of uncertainty analysis and linear regression was carried out); (2) comparison of the AB with the reference gravimetric method (in [6], the Mann–Whitney test and the Spearman’s correlation were used, also, a regression analysis was performed in accordance with [7]); (3) the evaluation of possible error trends by using Bland–Altman plots [8,9]; and (4) the impact of meteorological variables on measurement methods (a multiple linear regression analysis was carried out between AB absolute errors and meteorological parameters that were measured at the sampling point). The uncertainty between a couple of ABs was calculated in accordance with [10].

In [11], robust statistics were applied to assess temporal trends in PM_2.5_, PM_10_, and PM_2.5_/PM_10_ ratios. In accordance with [11], robust statistics are those that are not-sensitive to non-normal distributions and to extreme values in both tails of the distributions. In [11], the ratios of PM_2.5_/PM_10_ were characterized to better understand the behavior and potential future impacts of PM_2.5_ in the United Kingdom. The robust statistics that were used for data analysis in [11] were the following: median, various percentiles (fifth, 25th, 75th, and 95th), and Theil–Sen trend analysis [12,13].

According to [14], the National Capital Territory (NCT) of Delhi, India, is one of the most polluted regions in the world. In Delhi NCT, the annual PM_2.5_ concentration levels exceed the annual National Ambient Air Quality Standard of India, which is equal to $40\;\mu g/m^{3}$, by more than 200% [14,15,16,17,18,19,20]. In addition, in [21], a land-use regression model was used to estimate the mean annual PM_2.5_ concentrations over Delhi.

The research work presented in [14] was focused on tracking pollution build-up during the dry season (typically from the end of September to the end of June of the subsequent year, in the Delhi National Capital Region (NCR)). Furthermore, in [14], an aerosol optical depth (AOD) dataset was used. This AOD dataset was generated at a resolution of 1 km×1 km. In order to do this, the multiangle implementation of atmospheric correction (MAIAC) algorithm [22,23] applied to moderate resolution imaging spectroradiometer (MODIS) data was used. In addition, PM_2.5_ was estimated from MAIAC-AOD by multiplying spatially and temporally varying conversion factors at a spatial resolution of $0.1^{0}\times 0.1^{0}$. Moreover, all 1 km^2^ PM_2.5_ concentrations that were bias-corrected within Delhi NCR were averaged for each day. Finally, in order to ensure enough samples for robust statistics, the analysis was presented at weekly scale by averaging over 7 days.

In [24], PM_2.5_ pollution process was analyzed using long-term PM_2.5_ observations in three Chinese cities. In addition, the trend of the monthly mean of PM_2.5_ concentrations rising rates and PM_2.5_ concentrations using a robust Theil–Sen estimator was calculated.

In order to study the source and origin of ambient PM_2.5_ concentrations at a traffic site in Delhi, persistence analysis and nonparametric wind regression (NWR) were used in [25]. The persistence analysis was used to detect significant correlation between adjacent points and to infer the nature of sources that contribute to air pollution by PM_2.5_ concentrations in an area. Moreover, NWR analysis was performed to infer the nature of the sources of PM_2.5_ concentrations.

Nonparametric methods were also used in [26] to assess the influence on air quality of four industrial facilities that burn hazardous waste in southeast Kansas, Unites States of America. In [26], factors that affect spatial and temporal distribution of PM_10_ and PM_2.5_ concentrations in the region were investigated. Moreover, the effect of wind direction on the above-mentioned distribution was investigated as well. In [26], the Kruskal–Wallis test was used for the following purposes: (i) to determine whether the sampling site and the sampling date were statistically significant factors for PM_10_ and PM_2,5_ concentrations; (ii) to check whether there were significant differences between the cities under analysis; and (iii) to check whether the effect that wind direction had over PM_10_ and PM_2.5_ concentrations was statistically significant. In addition, the effect of the targeted sources on PM_10_ and PM_2.5_ concentrations was analyzed by using the Wilcoxon-signed rank test.

The Kruskal–Wallis test was also used in [27] to test whether there were significant differences between some stations of the Athens Subway System, Greece. In short, the research work presented in [27] was aimed at studying the indoor environmental quality inside the natural ventilated and air-conditioned train cabins and platforms of four main stations of the Athens Metro.

Moreover, another example in which an application of the Kruskal–Wallis test can be found is [28]. In [28], this test was used to assess differences in PM_2.5_ concentration levels in Beijing, China. In short, the study presented in [28] was aimed at detecting spatiotemporal change patterns of PM_2.5_ concentrations and evaluating the relation between PM_2.5_ concentration and meteorological factors.

In [29], a time-series study was carried out to evaluate the association between daily PM_2.5_ concentrations and respiratory deaths in eight municipal districts in Beijing, using a generalized additive mixed model. In addition, as there was very little information about the time trend in mortality, a Bayesian model averaging method was used to develop a robust predictive performance.

In order to know the factors that affect citizens because of their exposure to NO_2_ and NOx (nitrogen oxides) produced by traffic, [30] was aimed at estimating the way in which the gradient near the road varies owing to changes in meteorological and ambient variables, and also due to traffic conditions. The study presented in [30] was performed in Las Vegas, USA. In [30], the authors employed generalized additive models to model the slopes of functions that were used to characterize the zones in which concentrations of NO_2_ and NOx were assessed. Furthermore, the estimation of the concentration of NO_2_ and NOx on the road was performed by using the ordinary least-squares regression method. The studies conducted in [30] could be used to better understand the effects that roads that are heavily traveled and that are adjacent to sidewalks and houses have on people. All this helps to achieve a better urban design and to better predict the level of exposure that people have to certain variables that are harmful to human beings.

In addition, in order to study fine-scale variations of PM_2.5_ concentrations and black carbon, in [31], “a three-point synchronous observation experiment” was carried out. Such an experiment was performed in Shanghai, China. Moreover, a characterization of concentrations of PM_2.5_ and black carbon was performed in [31], and a generalized additive model was used to show the relationship that PM_2.5_ concentrations and black carbon have with multiple factors. The results of [31] could contribute to the development of air pollution control strategies at roadsides.

In [32], in order to carry out indoor air quality predictions, a methodology based on modeling was presented. The predictions were made using artificial neural networks and the Personal-Exposure Activity Location Model [33,34]. The approach presented in [32] can be employed to determine exposures that urban workers have to air pollutants. The study presented in [32] was performed in Dublin, Ireland. In addition, in [35], a research work on particulate matter produced by “vehicle emissions at traffic intersections of street canyons in Hong Kong” was presented. In [35], detrended fluctuation analysis and autocorrelation analysis were used to study the distinguishing features of the concentration of particulate matter.

Furthermore, in [36], a model consisting of a wavelet neural network and a genetic algorithm was used to predict CO and PM_2.5_ concentrations. The above-mentioned model was developed to perform the estimation of concentrations of air pollutants in Shanghai. In addition, in [37], both parts of the urban park La Carolina (Quito, Ecuador) and some of the surrounding streets were modeled as random variables, which represented routes made in some areas to measure the concentration of PM_2.5_ in them. Then, nonparametric statistical inference techniques were used to classify the level of air pollution that the areas under study had, based on air quality indexes defined by the city of Quito [38]. In [37], a Kruskal–Wallis test was applied to test whether the above-mentioned random variables came from the same statistical population, and the Wilcoxon signed-rank test was used to perform the classification process in accordance with Quito air quality indexes [38]. The results of [37] showed that the air pollution level because of PM_2.5_ concentrations in that urban park was not in alert level. However, there are important decisions that have to be taken by the city authorities in order to improve the air quality in the area under study.

For each statistical model, regardless of whether this is a location model, a scale model, a linear regression model, or a model of any other type, there are different robust statistical tools that increase the reliability and accuracy of modeling and/or data analysis [39,40].

The main objective of this article is to perform the robust statistical analysis of PM_2.5_ concentration measurements in La Carolina Park, which is one of the most important urban parks of Quito. In this article, in order to robustly estimate the location and scale parameters of the data, the following estimators were used: (i) for robust location estimation: α-trimmed mean, trimean, and median estimators; and (ii) for robust scale estimation: median absolute deviation, semi interquartile range, biweight midvariance, and estimators based on a subrange [39,40,41,42].

The use of several of the above-mentioned estimators within the context of the analysis of PM_2.5_ concentrations in urban parks is novel and has its importance in the fact that with few data and without the need for them to fit a normal distribution, or some kind of parametric distribution, the central tendency and dispersion of the data can be estimated in a robust way. Thus, small variations in the data have no effect on their estimation.

In this article, in order to be able to understand characteristics of the type of data that were analyzed, the first thing that was done was to establish the selection criteria of the study area (i.e., La Carolina Park, Quito). Second, a statistical summary of the data was performed, and moments of first, second, third and fourth order were found. Moreover, the median and range of the data were found. Also, in order to reduce the effect that each data point could have, graphs of the data were shown, and attempts were made to soften the data under analysis. In addition, empirical confidence intervals for the median of the data were obtained with a 95% confidence level, and nonparametric confidence intervals for the median of the data were also obtained with a 95% confidence level.

Here, the air pollution levels of each variable under study were classified in accordance with the categories established by the city of Quito [38]. After that, the robust statistical analysis of location parameters and scale parameters of the data was performed. The results of this article are compatible with [37].

This article is structured as follows: The criteria that were taken into account to select the study area are explained in Section 2. The description of the PM_2.5_ measurement instrument that was used in this article and data collection process are given in Section 3. Section 4 is devoted to carry out the robust statistical analysis of the data. Finally, the conclusions are given in Section 5.

## 2. Selection Criteria of the Study Area: La Carolina Park

One of the fundamental criteria that was taken into consideration to choose La Carolina Park to carry out our study was that this urban park offers an extremely important environmental service for Quito. This urban park is an air pollution filter. However, the characteristics of the park and its surroundings are different from each other.

Therefore, taking into consideration what has been said above, this article is focused on studying the area of La Carolina Park that possibly has the highest levels of PM_2.5_ concentrations.

In order to select the study area of the park, the following four criteria were taken into account:

(A) Vehicular traffic.
(B) Number of public transportation routes.
(C) Woodland density.
(D) Land use.

**Figure 1:**
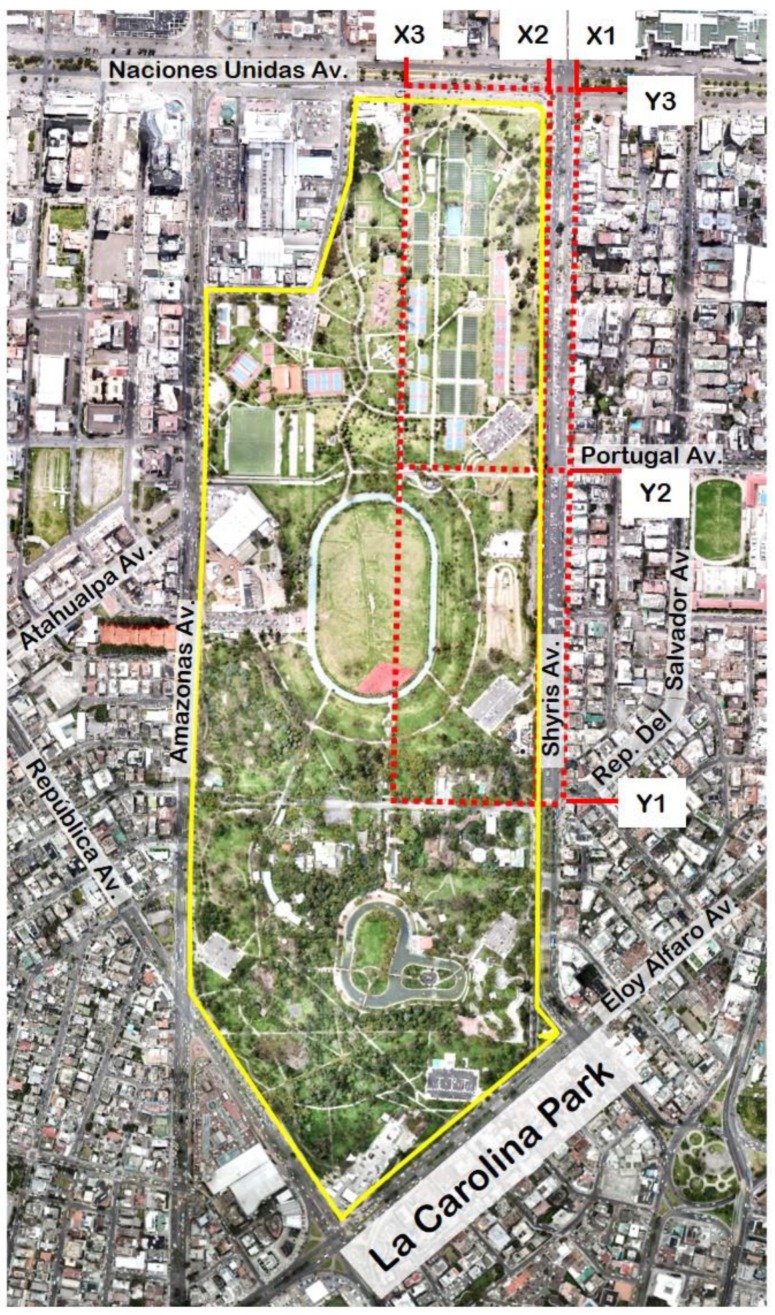
Park and route that was followed to perform the measurements. Urban park: Space delimited by the yellow lines; Route: Red dashed lines.

**Figure 2:**
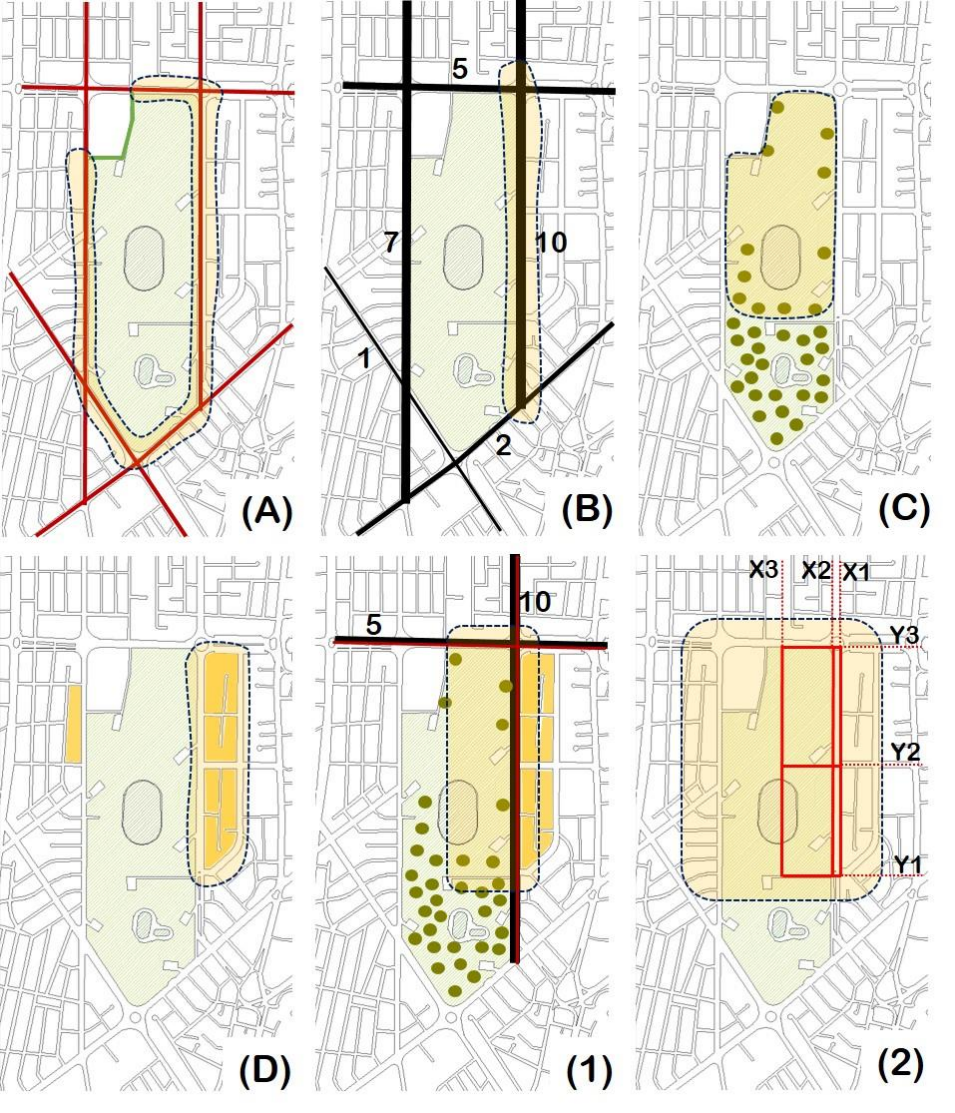
Selection criteria of the study area.

Furthermore, with the purpose of performing the experimental measurements, a route was followed through the park and its surroundings. The route that was followed is shown in Figure 1. In addition, this figure shows the six random variables ($X_{1},X_{2},X_{3},Y_{1},Y_{2},Y_{3}$) that refer to the routes along which the specific measurements were performed. The above-mentioned four selection criteria are shown in Figure 2, and the explanation of Figure 1 and Figure 2 is given below.

The above-mentioned six random variables, which refer to the routes along which the PM_2.5_ concentration measurements were performed, are shown in Figure 1 and represent the following:

- $X_{1}$ is a variable that is used to refer to the route that was followed through the sidewalk that is in front of La Carolina Park and on Avenida de los Shyris (shown as Shyris Av. in Figure 1).
  Measuring PM_2.5_ concentrations on this sidewalk is important, because on this sidewalk, the greatest commercial activity that exists around the park can be found. On this sidewalk, there are many high-rise buildings with shopping centers, companies, bus stops, homes, and entertainment venues, among other things, which act as barriers that prevent a satisfactory dissipation of PM_2.5_ concentrations. Therefore, the passers-by that walk on this sidewalk are exposed to the air pollution that is generated by the traffic of cars and buses that circulate along Avenida de los Shyris. In addition, the polluted wind that collides with buildings on this sidewalk bounces and creates whirls, which carry polluted air and impact the bodies of pedestrians.
- $X_{2}$ is a variable that is used to refer to the route that was followed through the sidewalk that is on the border that separates La Carolina Park from Avenida de los Shyris.
  Measuring PM_2.5_ concentrations on this sidewalk is important, because people walk along this sidewalk to have more contact with green spaces. On this sidewalk, there are also bus stops, and people use it to go jogging and do other activities.
- $X_{3}$ is a variable that is used to refer to the route that was followed through the imaginary line that crosses the park center and that is parallel to $X_{1}$ and $X_{2}$.
  Measuring PM_2.5_ concentrations on this imaginary line is important, because it represents a region with trees, vegetation, and green spaces. Moreover, this region has no bus or car traffic. In addition, this region has many areas that have been specifically designed for citizens to practice outdoor sports in the park.
- $Y_{i}$ is a variable that is used to refer to the route that was followed through Avenida República del Salvador (shown as Rep. Del Salvador Av. in Figure 1) and a path through the park that is an extension of Avenida República del Salvador. This path is used by people to go to the park center.
  The observation of this variable is important, because it takes into account parts of the park that have many trees and abundant vegetation.
- $Y_{2}$ is a variable that is used to refer to the route that was followed through Avenida Portugal (shown as Portugal Av. in Figure 1) and a path through the park that is an extension of Avenida Portugal. This path is used by people to go to the park center.
  This variable represents an imaginary line that is perpendicular to $X_{1}$, $X_{2}$, and $X_{3}$, and parallel to $Y_{1}$. Furthermore, the observation of this variable is important, because it takes into account parts of the park that have both very few trees and very little vegetation. However, this region has many areas that have been specifically designed for citizens to practice outdoor sports in the park.
- $Y_{3}$ is a variable that is used to refer to the route that was followed through Avenida Naciones Unidas (shown as Naciones Unidas Av. in Figure 1), which is a significant pollution source. This fact justified the need to consider this variable for the study that was carried out in this article. For this variable, the PM_2.5_ concentration measurements were performed on the sidewalk that is on the border that separates the park from Avenida Naciones Unidas.

The explanation of what is indicated in Figure 2A–D, is as follows:

- Figure 2A corresponds to the selection criterion (A) (i.e., Vehicular traffic), and this figure was made with the intention of showing that the five main streets that border the park have high traffic (indicated in red). This area is part of the financial and commercial heart of the city. Therefore, it has a proven ability to attract a high volume of private vehicles. Only the street that borders the park at the northwest corner has low traffic (indicated in green), which is because it is a street that does not direct traffic to places that are of great interest. In short, it is not a high traffic street. This part is generally used as a parking area.
- Figure 2B corresponds to the selection criterion (B) (i.e., Number of public transport routes), and this figure was made with the intention of showing that the streets that border the park have public transport lines, which in Quito is one of the most polluting types of vehicles. However, the two streets that connect the city in a north–south direction (i.e., Avenida Rio Amazonas, which is shown as Amazonas Av. in Figure 1, and Avenida de los Shyris) have seven and 10 bus lines, respectively; followed by Avenida Naciones Unidas, which has five bus lines. The junction at which Avenida de los Shyris and Avenida Naciones Unidas intersect is the one that concentrates most buses. In addition, it is possible that this junction has most air pollution emissions that come from the bus traffic.
- Figure 2C corresponds to the selection criterion (C) (i.e., Woodland density), and this figure was made with the intention of showing that forest density (represented by green markers) is a key factor in improving air quality. Therefore, this criterion focused on the identification of the park area with the lowest tree density. This would allow the identification of the most critical area, since having fewer trees may indicate that there are fewer natural systems that reduce pollution. In this sense, the northern part of the park was the one that met this criterion. This area is associated with physical activities such as exercise and sports in spite of its lack of trees and plants. This was a willful decision to create sports facilities rather than high-density green spaces. These facilities consist of the following: volleyball courts, basketball courts, and soccer fields.
- Figure 2D corresponds to the selection criterion (D) (i.e., Land use), and this figure was made with the intention of showing that, last but not least, the study should take into account people exposed to air pollution levels. Therefore, land use was estimated, especially on the ground floor of the buildings and commercial areas of the region that borders the park. To try to satisfy this criterion, the blocks that border the park and that have both a greater amount and diversity of activities on the ground floor were identified. The shaded areas in orange were the ones that were chosen, according to this criterion. In addition, the orange areas located on Avenida de los Shyris were the ones that showed the greatest amount and diversity of commercial premises. Furthermore, it was identified that the above-mentioned blocks contained the highest percentage of food stores on the ground floor. In addition, the enclosures of these food stores do not have any filter mechanism for the air pollution that may be generated on this street.

It should also be mentioned that there are car parking areas within the park. However, although these parking areas are distributed equally throughout the park, with four areas to the south and four areas to the north, the difference in vegetation density between them is a factor that must be taken into account.

As a result of the above-mentioned four overlapping criteria, it was observed that the zone most prone to conditions of higher levels of air pollution and where people would be more exposed by the activity they perform is the zone that is indicated in Figure 2 (1) (i.e., Avenida de los Shyris and Avenida Naciones Unidas)—that is, the zone that is located in the northeast of the park. Therefore, this fact justified the need to measure air pollution in that zone.

According to the four selection criteria that were mentioned at the beginning of this section, Figure 2 (2) shows the routes that were followed to perform the PM_2.5_ concentration measurements within the most critical area of the park.

## 3. PM_2.5_ Measurement Instrument and Data Collection Description

In this article, a portable CEL-712 Microdust Pro monitor pair with a GPS (Global Positioning System) device was used to perform the PM_2.5_ concentration measurements. This measurement instrument was calibrated by CASELLA [43]. The calibration results were the following:

- Instrument type: Microdust Pro (Standard Range: $0–2.5,\;0–25,\;0–250,\;0–2500\;\mathrm{mg}/m^{3}$).
- Serial Number: 4111986.
- Calibration Principle: Calibration was performed using an ISO 12103 Pt1 A2 Fine test dust (Natural ground mineral dust, predominantly silica, Arizona Road Dust equivalent, Particle size range 0.1 to 80 μm). A Wright Dust feeder system was used to inject and disperse calibration dust within a wind tunnel system. Particulate mass concentration was established using isokinetic sampling and gravimetric methods.
- Calibration Results Summary:
  ○ Applied concentration: $4.94\;\mathrm{mg}/m^{3}$.
  ○ Indication: $4.91\;\mathrm{mg}/m^{3}$.
  ○ Error: −1%.
  ○ Target Error <15%.

In this article, the sampling time of the CEL-712 Microdust Pro monitor was 1 s, and the measurements were averaged to show the results each 10 s. Moreover, the GPS saved the coordinates of the route each 1 min.

The above-mentioned equipment were used to carry out their measurements at a height of 1.5 m, placing the particle inlet forward. Here, the walking speed was approximately equal to 2 km/h. Then, in order to build a pollution map in QGIS software, both collected and GPS data were used. In this research work, PM_2.5_ concentration measurements were performed in October 2018 and January 2019. Moreover, all measurements were performed during the morning rush hours (8:00–10:00), as it is a time of the day with the worst air quality conditions, due to the low planetary boundary height and reduced atmospheric mixing.

According to [44], the CEL-712 Microdust Pro has the highest measurement range of any occupational dust measurement instrument available on the market. In addition, due to the large data storage capacity of this equipment, up to 500 measurement results can be taken and stored. Moreover, the user can generate reports in an easy way using the intuitive report wizard. The key features of this instrument and applications can be found in [44]. A characteristic of the CEL-712 Microdust Pro that was important in order to carry out the research work presented in this article is that this instrument can be used for spot checks and walk-through surveys. In addition, this instrument has the advantage that the user can instantly see when and where excessive dust levels are occurring [44].

In accordance with [44], another characteristic that this instrument has, which makes it unique because no other device has it, is that the CEL-712 Microdust Pro uses an on-site calibration filter to provide a spot check of the linearity of the instrument.

**Figure 3:**
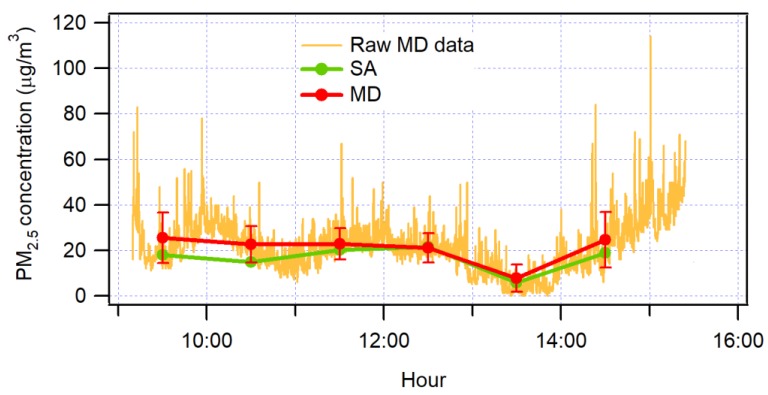
Raw Microdust (MD) Pro 10 s data (orange line), plotted against city air quality network Thermo Fisher Scientific BAM (SA) hourly data and hourly Microdust Pro data (MD).

Furthermore, the CEL-712 Microdust Pro was validated by collocation following the instructions of the U.S. Environmental Protection Agency (U.S. EPA) to evaluate low-cost sensors [45]. For the case under study, the U.S. EPA also recommends [46,47]. For the case under analysis, the CEL-712 Microdust Pro was validated by collocation with the Thermo Fisher Scientific BAM equipment of the Air Quality Network station of Quito, run by the Environmental Protection Agency (in Spanish, it is called “Secretaria de Ambiente” (SA)) of Quito, for six hours. The experiment time had to be limited to six hours, due to the battery lifetime, and the fact that the battery could not be replaced because the monitoring station was located in a school. Figure 3 shows the raw Microdust Pro data and hourly averages of Microdust Pro and the BAM method. It can be seen that while there is a variation in the Microdust Pro data, BAM data fits in the one standard deviation of the Microdust Pro data. In accordance with [48], the validation was performed at times were the relative humidity is low [49].

Before finishing this section, it is important to mention that studies aimed at conducting the evaluation and comparison of several PM_2.5_ monitors can be found in [6]. In addition, research articles focused on the calibration and validation of low-cost particulate matter sensors can be found in [4,48].

## 4. Robust Data Analysis

This section is divided into three parts. First, a statistical summary of the data is presented. Second, a nonparametric inferential analysis is performed. Finally, the section ends using robust estimators to analyze measures of centralization and dispersion of the data of the variables under study. The latter is justified by the fact that robust estimators are relatively insensitive to small changes in the data.

### 4.1. Statistical Summary of the Dataset

In accordance with [50], in this research work, there is a set of data that is going to be analyzed in order to properly interpret the relationships between them. Furthermore, in this subsection, different types of graphs will be used with the intention of showing a simple transmission of the analysis and conclusions. Here, it has been considered that these graphs will allow a simple comparison between the data and, on the other hand, highlight differences between the variables under study. According to [51], these graphs make a clear and precise representation of the consequences.

**Figure 4:**
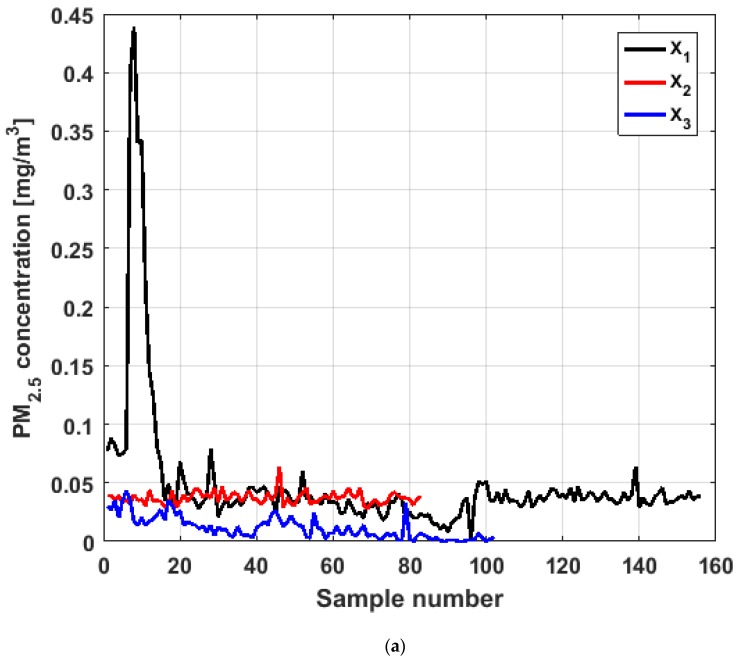

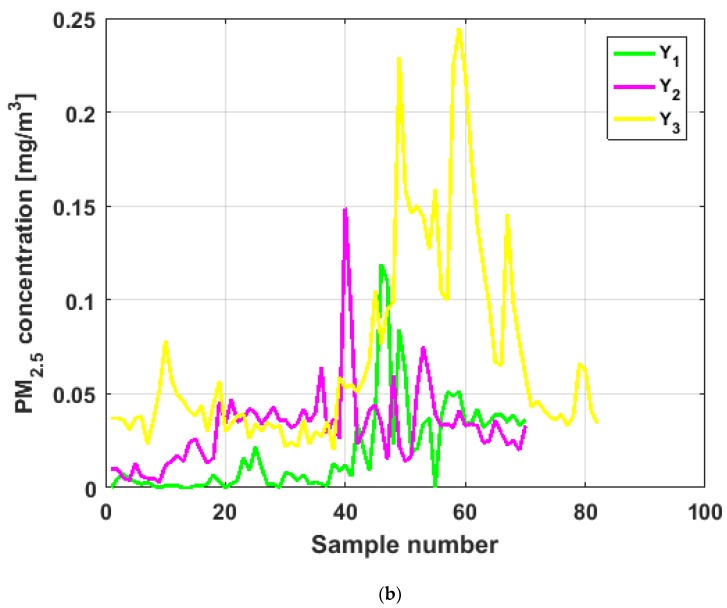
Graphs of the data obtained during the data collection process: (**a**) PM_2.5_ concentration measurements of $X_{1}$, $X_{2}$, and $X_{3}$; (**b**) PM_2.5_ concentration measurements of $Y_{1}$, $Y_{2}$, and $Y_{3}$.

To perform the analysis, a graph of each of the datasets was first considered, where these were treated as if they were a time series (see Figure 4). The order in which the data was collected is represented on the abscissa axis, while the value of the data is represented on the axis of the ordinates.

Figure 4 shows the collected data separated by the lines that are parallel to the park, which are represented by the letter X, and the lines that are perpendicular to the park, which are represented by the letter Y (see Figure 1 and Figure 2). The results of the PM_2.5_ measurements that are represented in this figure are the observations of the variables represented in Figure 1, which have been explained in Section 2.

From Figure 4, it can be seen that the variable $X_{1}$, which is the one with the most data, and the variable $Y_{3}$ are the ones with the greatest fluctuations, while the variables $X_{2}$ and $X_{3}$ behave in a more linear way and with less variations, although the values of $X_{3}$ are lower than those of $X_{2}$. The other two variables, $Y_{1}$ and $Y_{2}$, apparently behave analogously to each other and different from the rest. Below, a statistical summary of the data is shown in Table 1. This table includes measures of central tendency, variability, and shape.

When comparing the summary statistics presented in Table 1 with those presented in [6], it can be said that the difference between both studies is that in [6], the skewness and kurtosis were not studied. However, in this article, it is important to study these third and fourth moments, because it is important to analyze whether the data comes from heavy-tailed distributions. According to [40], heavy-tailed distributions are those probability distributions whose density tails are not bounded by the normal density tails.

**Figure 5:**
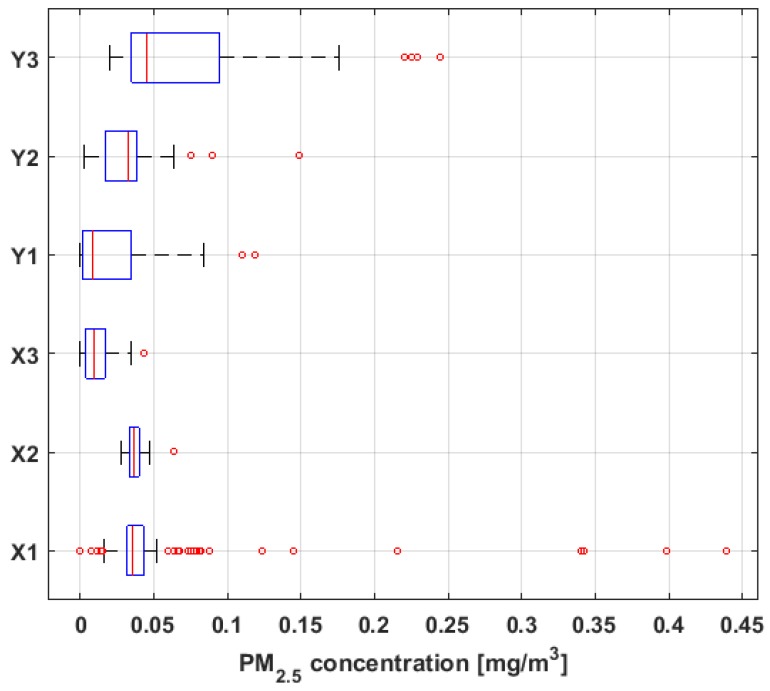
Multiple box-plot of data.

**Figure 6:**
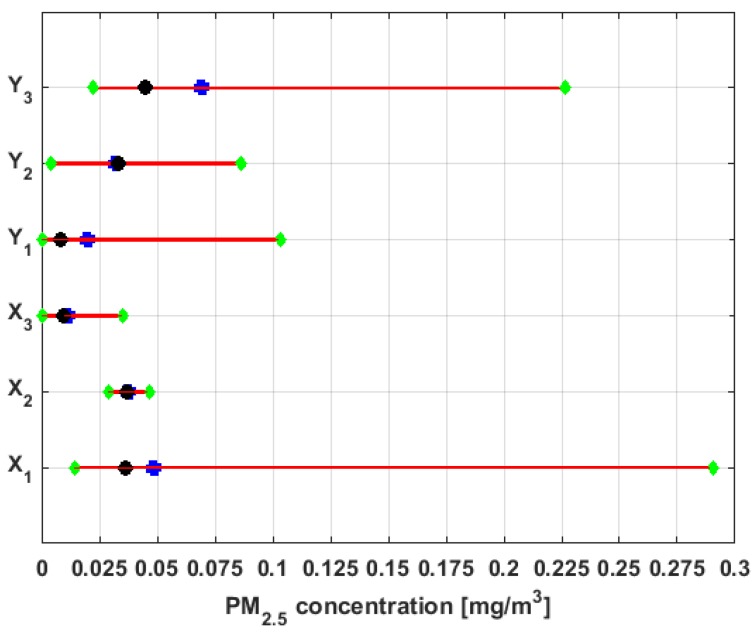
Empirical confidence intervals for the median with a confidence level of 95%. Median: Black circle; Mean: Blue square; Ends of the intervals: Green diamond.

Figure 5 shows the multiple box-plot of data from each variable, and Figure 6 shows the empirical 95% confidence intervals for the median [52], including both the mean and the median of the data.

Based on the previous figures, at first sight it is observed that the sizes of the six variables under study are different, with $X_{1}$ having almost double the observations compared to each of the rest of the variables. In addition, it is observed that the means are higher than the medians in all cases, except for $Y_{2}$. The aforementioned indicates the existence of extreme values on the right, which is corroborated by the multiple box-plot shown in Figure 5.

In addition, the values of the standard deviation shown in Table 1 confirm that the variability of $X_{1}$ is similar to the variability of $Y_{3}$, that the dispersion of the values of $X_{2}$ and $X_{3}$ is small, and that there is some similarity between the fluctuations of $Y_{1}$ and the fluctuations of $Y_{2}$.

Moreover, it can be seen that all the variables present a great lack of normality due to the high skewness values. All the skewness values shown in Table 1 are positive, so the distributions are lengthened to the right. The lack of normality is reaffirmed for most of the variables, since almost all the kurtosis values are much greater than 3, in particular the kurtosis values of $X_{1}$ and $Y_{2}$.

Next, in order to see the trends of the variables under study, the technique of moving average (MA) was used [53]. The MA technique was used to soften the data of the time series, in order to reduce the influence that each individual data had.

**Figure 7:**
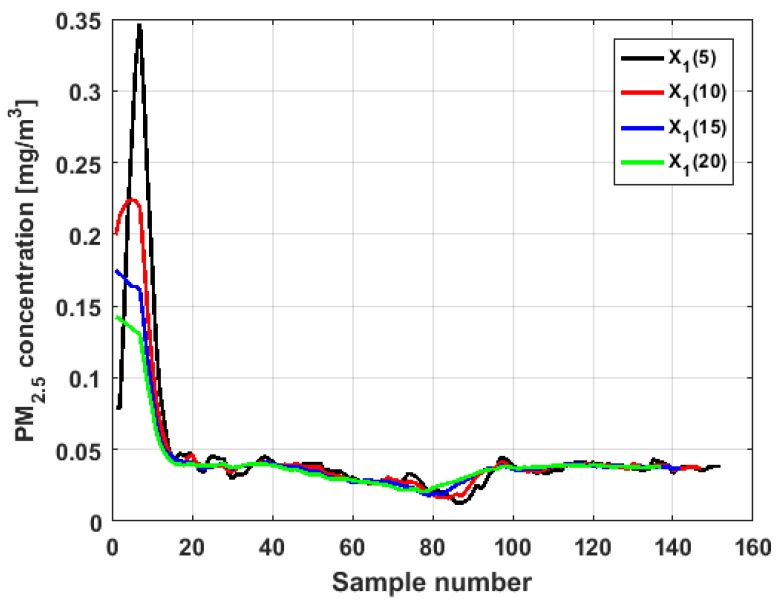
Moving average (MA) of $X_{1}$ for different sizes.

**Figure 8:**
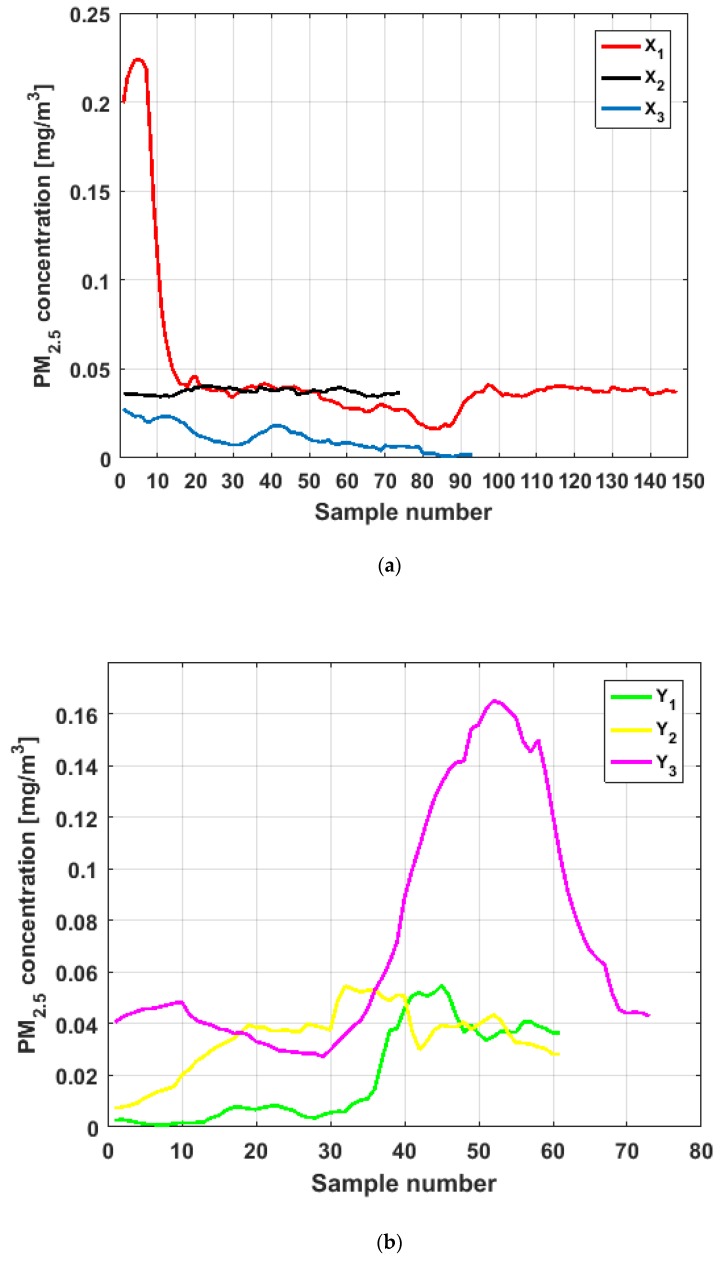
MA of size 10 of the data obtained during the data collection process: (**a**) MA of $X_{1}$, $X_{2}$, and $X_{3}$; (**b**) MA of $Y_{1}$, $Y_{2}$, and $Y_{3}$.

In this article, MA of size 5, 10, 15, and 20 were considered. However, after verifying that for each variable the MA technique behaved in the same way for each of the above-mentioned sizes, because the ends were maintained and the curves softened very little when the data were stable, it was decided to select the MA of size 10 to find representations analogous to those in Figure 4. Figure 7 shows the application of the MA of different sizes to the variable $X_{1}$, and the graphs of the application of the MA of size 10 to the six variables are shown in Figure 8.

When analyzing Figure 7 and Figure 8, it can be said that the conclusions are the same as for Figure 4. Specifically, the major fluctuations occur in the variables $X_{1}$ and $Y_{3}$, although these fluctuations occur by changing the trend. Moreover, the trend changes do not occur in specific moments, but rather in time intervals. The similarity between the variables $X_{2}$ and $X_{3}$ is repeated, where $X_{3}$ has lower values. In addition, the similarity between $Y_{1}$ and $Y_{3}$ and its difference with the remaining variables are also repeated.

### 4.2. Nonparametric Inferential Statistical Analysis

In this part of the article, the first objective is to know if all the variables can be considered as samples that come from six continuous random variables with distribution functions $F_{X_{1}}\left(x\right)$, $F_{X_{2}}\left(x\right)$, $F_{X_{3}}\left(x\right)$, $F_{Y_{1}}\left(x\right)$, $F_{Y_{2}}\left(x\right)$, and $F_{Y_{3}}\left(x\right)$, respectively. The location model for these samples can be established as that the six distribution functions are identical to the random variable Z, whose distribution function is F(x), and that the six distribution functions are $F\left(x-\theta_{1}\right)$, $F\left(x-\theta_{2}\right)$, $F\left(x-\theta_{3}\right)$, $F\left(x-\theta_{4}\right)$, $F\left(x-\theta_{5}\right)$, and $F\left(x-\theta_{6}\right)$, respectively, in which $\theta_{i}$ (for i=1,…,6) is a location parameter, and will be assumed to be an order statistic.

If the six samples come from populations that have a common median, a statistical hypothesis test will be established and confidence intervals will be obtained, both bilateral, to compare whether the six variables have sufficiently different medians. In addition, it will be analyzed whether the cause of these differences between the medians can be attributed to chance, or to another cause [54,55,56].

In this article, observations were carried out on different groups of variables, which will be considered to be independent of each other, because they are values that come from different places [54].

Here, in accordance with [54,55,56], it was considered the test where the null hypothesis was:

$$H_{0}:Median=M_{0},$$

against the alternative hypothesis:

$$H_{1}:Median\neq M_{0}.$$

Taking into account that the variable is continuous, assuming that the null hypothesis is true and that the sample data are consistent with the median value, half of the observations will be less than $M_{0}$, and the other half will be greater.

The test statistic will be K, which represents the number of sample observations greater than the $M_{0}$ value, and although the order statistics with index r, $X_{\left(r\right)}$ of a sample do not have the same distribution as the original variable and are not independent of each other, it does happen that the order statistics follow binomial distributions.

For the case under study, K will be a binomial random variable of parameter h, which is the number of observations, and have a probability of success equal to 1/2, $K\sim Bin\left(h,\frac{1}{2}\right)$. Therefore, the null hypothesis, $H_{0}$, will be rejected if the test statistic, K, takes values greater than a certain constant, $K\geq k_{\frac{\alpha }{2}}$, or takes values less than another constant, $K\leq k_{\frac{\alpha }{2}}^{\prime }$, where α is the significance level.

If bilateral confidence intervals are chosen, these intervals will be of the form $\left(X_{\left(k_{\frac{\alpha }{2}}^{\prime }\right)},X_{\left(k_{\frac{\alpha }{2}}\right)}\right)$. By choosing those values so that the probability on the right is equal to the probability on the left, it can be shown that these values verify the following:

1. $k_{\frac{\alpha }{2}}^{\prime }$ is the largest integer that verifies $\sum_{i=0}^{k_{\frac{\alpha }{2}}^{\prime }}\left(\begin{array}{c} N\\ i \end{array}\right)\frac{1}{2^{N}}\leq \frac{\alpha }{2}$,
2. $k_{\frac{\alpha }{2}}$ is the smallest integer that verifies $\sum_{i=k_{\frac{\alpha }{2}}}^{N}\left(\begin{array}{c} N\\ i \end{array}\right)\frac{1}{2^{N}}\leq \frac{\alpha }{2}$,
3. The *p*-value of the statistical hypothesis test is equal to $2\cdot min\left\{\sum_{i=0}^{K}\left(\begin{array}{c} N\\ i \end{array}\right)\frac{1}{2^{N}},\sum_{i=K}^{N}\left(\begin{array}{c} N\\ i \end{array}\right)\frac{1}{2^{N}}\right\}$,

where N is the number of independent trials.

**Figure 9:**
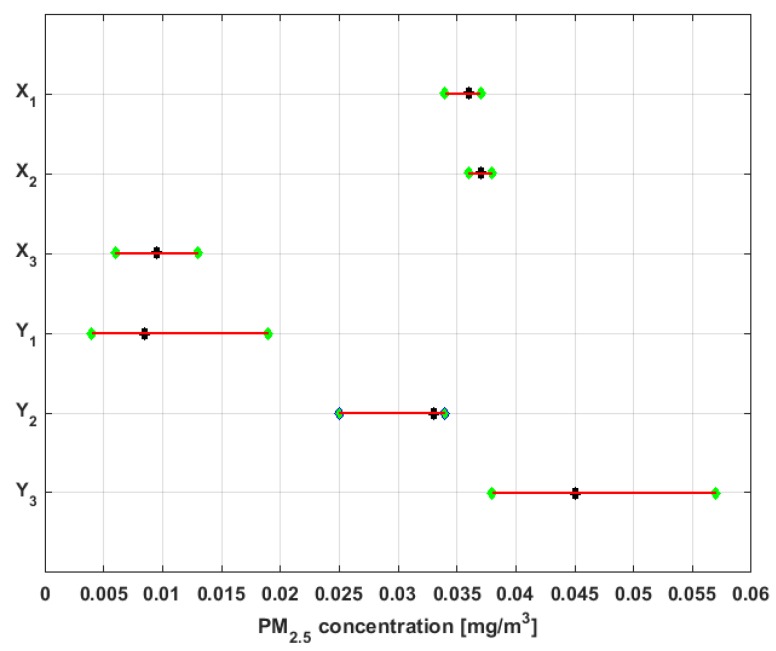
95% nonparametric confidence intervals for the medians. Median: Black circle; Ends of the intervals: Green diamond.

For the statistical hypothesis testing, where the null hypothesis was:

$$H_{0}:Median=M_{e},$$

where $M_{e}$ was the population median, and with a significance level α=5%, the lower and upper rejection limits, and length of the confidence interval were found. In addition, the *p*-value of the hypothesis test was found. In [6], it was considered that a *p*-value lower than 0.05 was statistically significant. In the present article, a *p*-value lower than 0.05 was also considered statistically significant, due to the fact that the rejection limits were calculated at α=5%. These results are shown in Table 2. Also, Figure 9 shows both the nonparametric confidence intervals for the medians of the variables, with a 95% confidence level [54,55,56], and the medians of the variables.

In view of these results, it can be said that the median of $Y_{2}$ is different from the median of any of the other variables, since the rejection limits include the rest of the medians. The same can be said of $Y_{3}$, because the rejection region of the hypothesis test for this variable includes the rest of the medians. In addition, the hypothesis that the medians of $X_{1}$ and $X_{2}$ can be the medians of the populations $X_{3}$ and $Y_{1}$ is rejected, and the hypothesis that the medians of $X_{3}$ and $Y_{1}$ are equal cannot be rejected. In addition, the hypothesis that the medians of the variables $X_{1}$ and $X_{2}$ are equal cannot be rejected.

The confidence intervals of smallest size are those corresponding to the variables $X_{1}$ and $X_{2}$, which indicates that these two variables are the ones that have less variability. On the other hand, the confidence intervals of greatest size are those of the variables $Y_{1}$ and $Y_{3}$. Therefore, it can be inferred that these variables have the greatest variability. Furthermore, the other two variables (i.e., $X_{3}$ and $Y_{2}$) have intervals of an intermediate length with respect to the length of the other intervals. Therefore, the variability of these last two variables will also be intermediate with respect to the variability of the other variables.

In accordance with the above explanation and Figure 9, the variables under study have been classified into four groups. One group consists of $Y_{2}$, another group consists of $Y_{3}$, a third group consists of $X_{1}$ and $X_{2}$, and the last group consists of $X_{3}$ and $Y_{1}$.

Next, the nonparametric hypothesis tests that were performed to test the category in which each of the six variables under study was located are going to be analyzed. In order to do this, the categories that are established by the Quito Air Quality Index (QAQI) for air pollution by PM_2.5_ [38] were taken into consideration.

In accordance with [38], for an average concentration of PM_2.5_ in 24 h, the air pollution categories are the following:

1. Interval for Desirable level of PM_2.5_ concentration: [0,0.025) $\mathrm{mg}/m^{3}$.
2. Interval for Acceptable level of PM_2.5_ concentration: [0.025,0.050) $\mathrm{mg}/m^{3}$.
3. Interval for Caution level of PM_2.5_ concentration: [0.050,0.150) $\mathrm{mg}/m^{3}$.
4. Interval for Alert level of PM_2.5_ concentration: [0.150,0.250) $\mathrm{mg}/m^{3}$.
5. Interval for Alarm level of PM_2.5_ concentration: [0.250,0.350) $\mathrm{mg}/m^{3}$.
6. Interval for Emergency level PM_2.5_ concentration: [0.350,∞) $\mathrm{mg}/m^{3}$.

**Figure 10:**
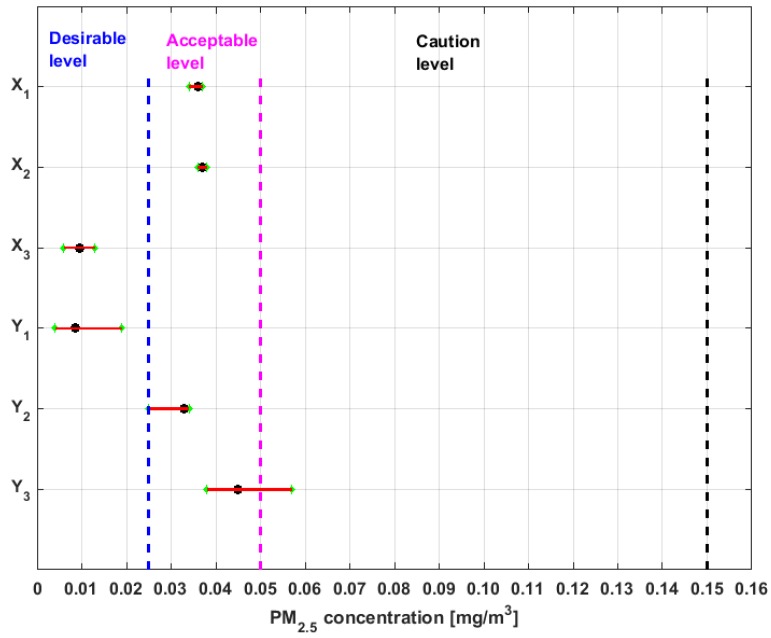
Medians, 95% nonparametric confidence intervals for the medians, and the bands that delimit the three lowest categories of air pollution by PM_2.5_ concentrations in Quito. Median: Black circle; Ends of the intervals: Green diamond.

The medians, 95% confidence intervals for the medians, and bands that delimit the three lowest categories of air pollution by PM_2.5_ concentration in which air quality in Quito is classified [38] are shown in Figure 10.

From Figure 10, it can be seen that the nonparametric confidence intervals for the median of $X_{3}$ and $Y_{1}$ are contained in the Desirable level. Therefore, the null hypothesis that the median of $X_{3}$ and $Y_{1}$ are at the Desirable level cannot be rejected at a significance level of α=5%. Moreover, it can be rejected the hypothesis that these medians belong to the other levels of air quality at the 95% confidence level.

Furthermore, the hypothesis that the medians of the variables $X_{1}$, $X_{2}$, and $Y_{2}$ are at an Acceptable level cannot be rejected, and it is rejected the hypothesis that these medians can belong to the other levels.

In addition, the hypothesis that the median of variable $Y_{3}$ can belong to the Acceptable level or Caution level cannot be rejected, but the hypothesis that this median belongs to the other four levels is rejected.

In addition, taking into account the moments of orders two, three, and four shown in Table 1, although $X_{2}$ has the same median as $X_{1}$, it does not come from the same distribution as $X_{1}$. Therefore, if $X_{3}$ and $Y_{1}$ are considered to be elements of the same group, it can be said that the null hypothesis that the distributions of the five groups of variables are different from each other is rejected.

**Figure 11:**
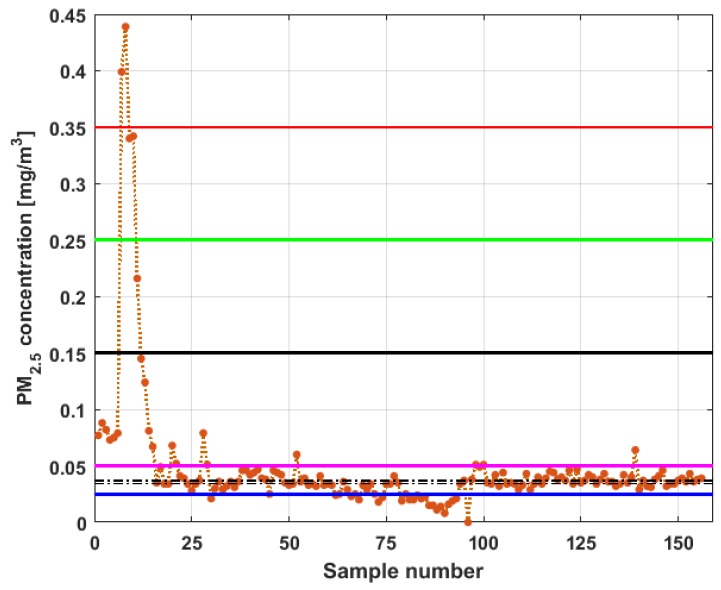
Observations of variable $X_{1}$, together with the 95% nonparametric confidence interval for the median (shown with dashed black lines) and the limits that define the categories of the level of air pollution by PM_2.5_ (Desirable level: Blue line; Acceptable level: Magenta line; Caution level: Black line; Alarm level: Green line; Emergency level: Red line).

**Figure 12:**
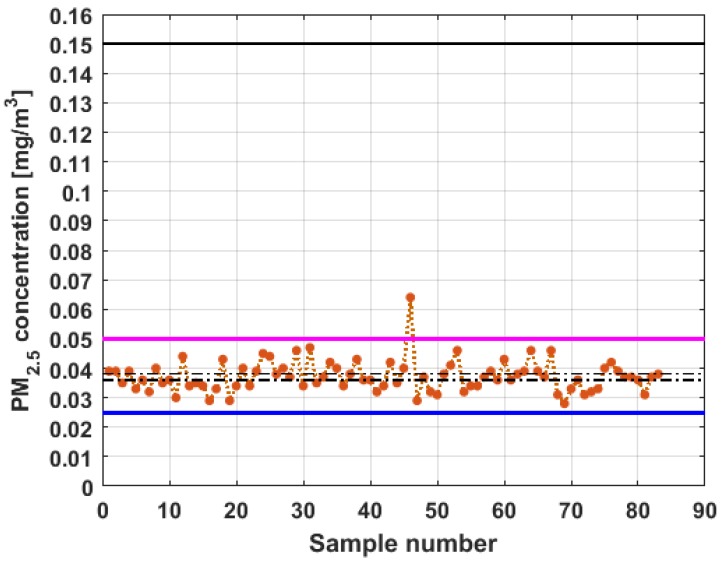
Observations of variable $X_{2}$, together with the 95% nonparametric confidence interval for the median (shown with dashed black lines) and the limits that define the categories of the level of air pollution by PM_2.5_ (Desirable level: Blue line; Acceptable level: Magenta line; Caution level: Black line).

**Figure 13:**
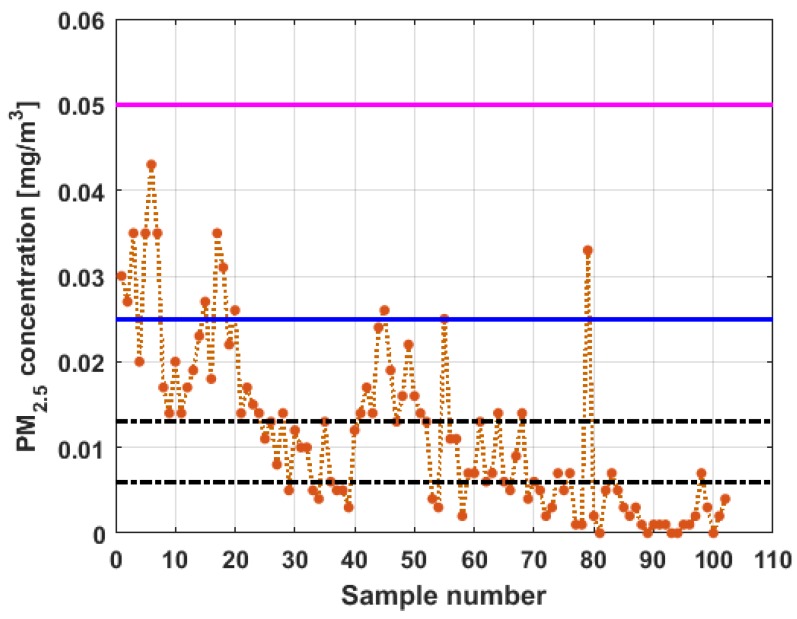
Observations of variable $X_{3}$, together with the 95% nonparametric confidence interval for the median (shown with dashed black lines) and the limits that define the categories of the level of air pollution by PM_2.5_ (Desirable level: Blue line; Acceptable level: Magenta line).

**Figure 14:**
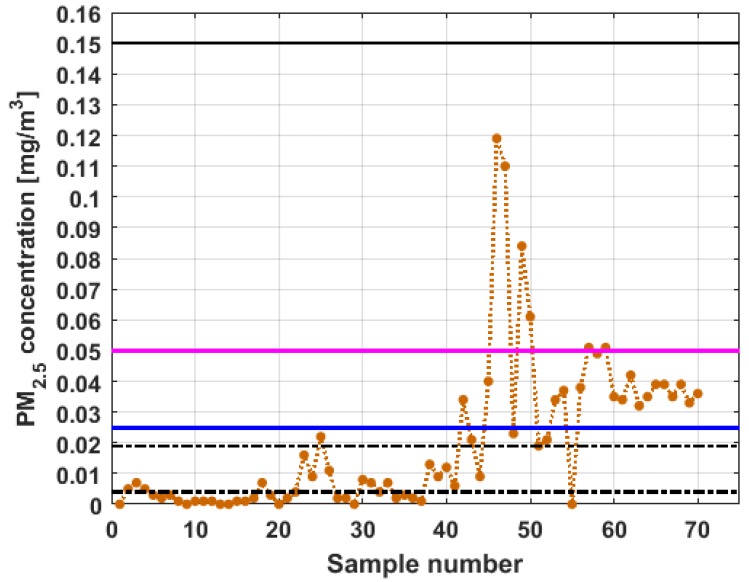
Observations of variable $Y_{1}$, together with the 95% nonparametric confidence interval for the median (shown with dashed black lines) and the limits that define the categories of the level of air pollution by PM_2.5_ (Desirable level: Blue line; Acceptable level: Magenta line; Caution level: Black line).

**Figure 15:**
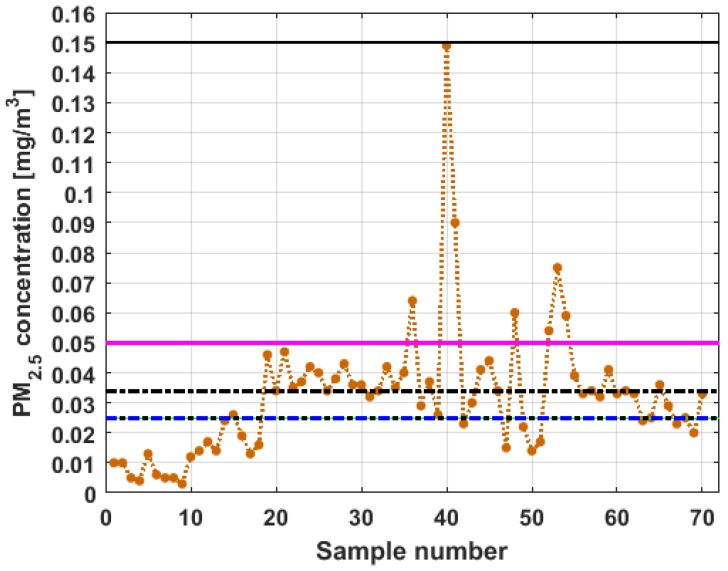
Observations of variable $Y_{2}$, together with the 95% nonparametric confidence interval for the median (shown with dashed black lines) and the limits that define the categories of the level of air pollution by PM_2.5_ (Desirable level: Blue line; Acceptable level: Magenta line; Caution level: Black line).

**Figure 16:**
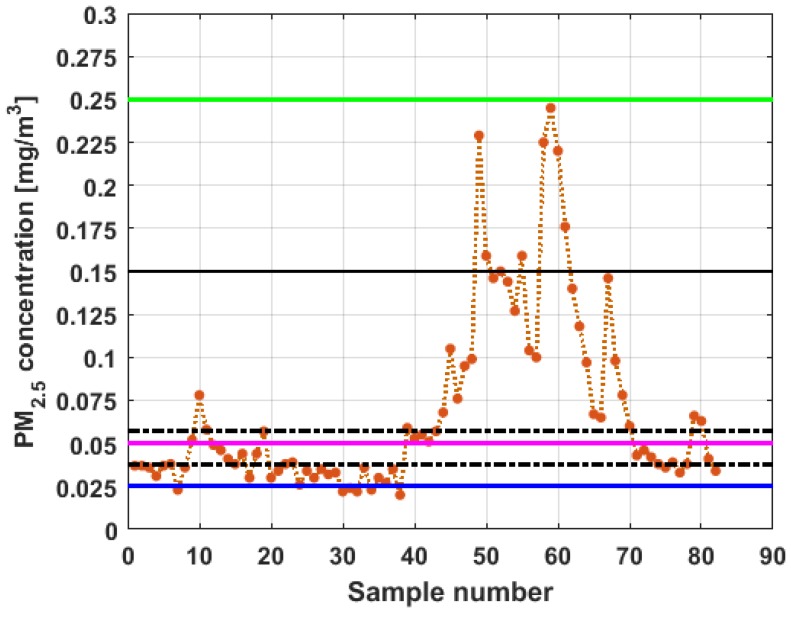
Observations of variable $Y_{3}$, together with the 95% nonparametric confidence interval for the median (shown with dashed black lines) and the limits that define the categories of the level of air pollution by PM_2.5_ (Desirable level: Blue line; Acceptable level: Magenta line; Caution level: Black line; Alarm level: Green line).

Figure 11, Figure 12, Figure 13, Figure 14, Figure 15 and Figure 16 show the observations of variables $X_{1}$, $X_{2}$, $X_{3}$, $Y_{1}$, $Y_{2}$, and $Y_{3}$, respectively, together with the 95% nonparametric confidence interval for the median and the limits that define the categories of the above-mentioned levels of air pollution by PM_2.5_ concentrations [38].

From Figure 11, Figure 12, Figure 13, Figure 14, Figure 15 and Figure 16, it can be said that the only variable that at any time exceeds all the air quality limits is $X_{1}$. In addition, this variable has many observations outside its nonparametric confidence band for the median, which is also a common characteristic that all the variables under study have. In addition, in terms of exceeding the air quality limits, there is the variable $Y_{3}$, which almost reaches the Alarm level, and indicates a significant variability.

With respect to $X_{2}$, it can be said that this variable practically does not exceed the Acceptable level. Furthermore, it can be said that $X_{3}$ sometimes exceeds the Desirable level; however, most observations of $X_{3}$ remain below the Desirable level.

Finally, it can be said that $Y_{1}$ exceeds the Acceptable level with few observations, and many of its observations are at the Desirable level, while a large part of the observations of $Y_{2}$ are above the Desirable level, and some exceed the Acceptable level, being below Caution level.

Before finishing this subsection, the six variables under study are going to be divided into two different groups, one consisting of the X variables and the other consisting of the Y variables. In addition, it will be said that the X variables are parallel to the park and some of them cross it in this way, while the Y variables are perpendicular to the park and some of them cross it in this way.

Therefore, it is going to be analyzed whether the air pollution by PM_2.5_ is more harmful for citizens when the park is crossed longitudinally or when it is crossed transversely. To this end, the Wilcoxon rank-sum test adapted to compare the above-mentioned two groups against a one-sided alternative is going to be used [54].

For this case, the null hypothesis is:

$$H_{0}:air\;pollution\;due\;to\;group\;Y=air\;pollution\;due\;to\;group\;X,$$

and the alternative hypothesis is:

$$H_{1}:air\;pollution\;due\;to\;group\;Y>air\;pollution\;due\;to\;group\;X.$$

Table 3 shows the count data—that is, the number of observations that respond in a certain manner to air pollution by PM_2.5_ concentrations. This table shows the multivariate frequency distribution of the variables.

For the case under study, the Wilcoxon rank-sum test statistic [54] for the Y sample is $W_{N}=11.5$, the expected value is $E\left(W_{N}\right)=10.5$, and the variance is $var\left(W_{N}\right)=4.475$. Therefore, the approximate *p*-value, using the normal approximation to the distribution of $W_{N}$ with continuity correction [54], is 0.4066. As a result, the null hypothesis that air pollution due to group Y is equal to air pollution due to group X cannot be rejected at a significance level of α=5%. Statistically speaking, the air pollution due to group Y is equal to the air pollution due to group X.

The difference between the nonparametric statistical tools used in [26,27,28] and the analysis performed in this subsection, is that in [26,27,28], the study focused on obtaining only estimates of the median of the analyzed data. To carry out the study that was presented in [26,27,28], the Kruskal–Wallis test and the Wilcoxon-signed rank test were used. However, in this subsection, a nonparametric statistical analysis procedure that was focused on obtaining measures to estimate the central tendency of the data and their dispersion was developed. The nonparametric statistical analysis procedure developed in this article not only estimates the median of the data, but also analyzes the variability of the data, and uses all this information to classify the variables under study according to established categories of air pollution levels [38].

### 4.3. Robust Analysis of Location Parameters

A parameter, θ, is called the location parameter for the random variable Ψ if the density function can be written as a function of ψ−θ. Therefore, the random variable Ψ−θ does not depend on θ. The location parameters usually indicate a value around which the bulk of the observations are grouped.

In this subsection, $\Psi_{1},\ldots ,\;\Psi_{n}$ was a sample and, in order to establish the estimators that were used, the sample order statistics [56] were considered: $\Psi_{\left(1\right)}\leq \Psi_{\left(2\right)}\leq \ldots \leq \Psi_{\left(n\right)}$. In addition, in order to find estimates where the distribution symmetry center can be found, some *L*-location estimators that are linear combinations of order statistics were considered. In short, the α-trimmed family [39,40] with 0≤α<0.5 given by Equation (1) was used. Moreover, other location estimators that will be considered are the mean and the median:

(1) $$\left(\alpha \right)=\frac{1}{n-2\left[n\cdot \alpha \right]}\sum_{i=\left[n\cdot \alpha \right]+1}^{n-\left[n\cdot \alpha \right]}\Psi_{\left(i\right)},$$

where […] denotes the integer part. In addition, the trimean [39,41] was used, which is given by Equation (2):

(2) $$TM=\frac{lower\;hinge+2\left(median\right)+upper\;hinge}{4}=\frac{Q_{1}+2Q_{2}+Q_{3}}{4},$$

where $Q_{i}$ is the *i*-th quartile.

**Figure 17:**
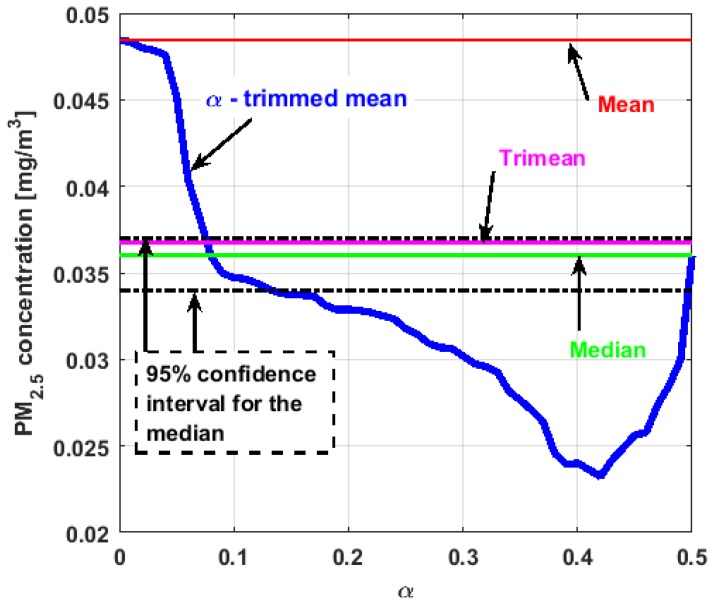
α-trimmed mean of $X_{1}$.

**Figure 18:**
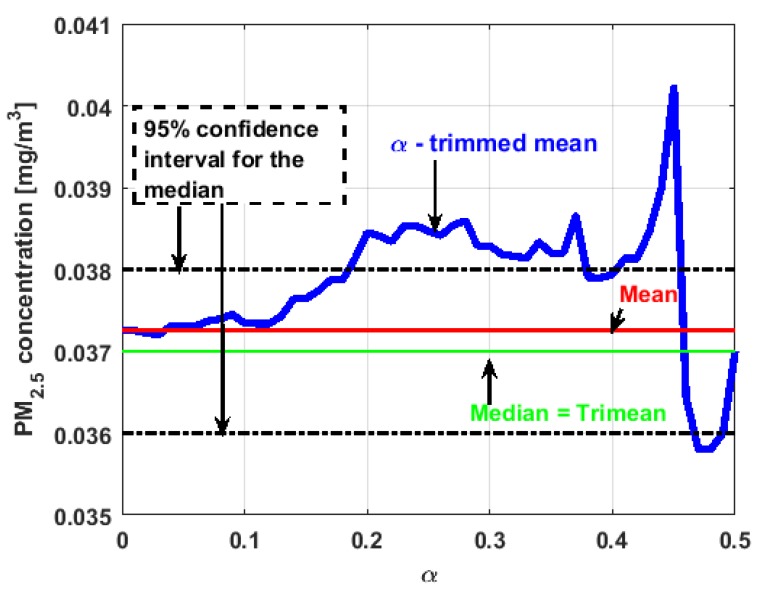
α-trimmed mean of $X_{2}$.

**Figure 19:**
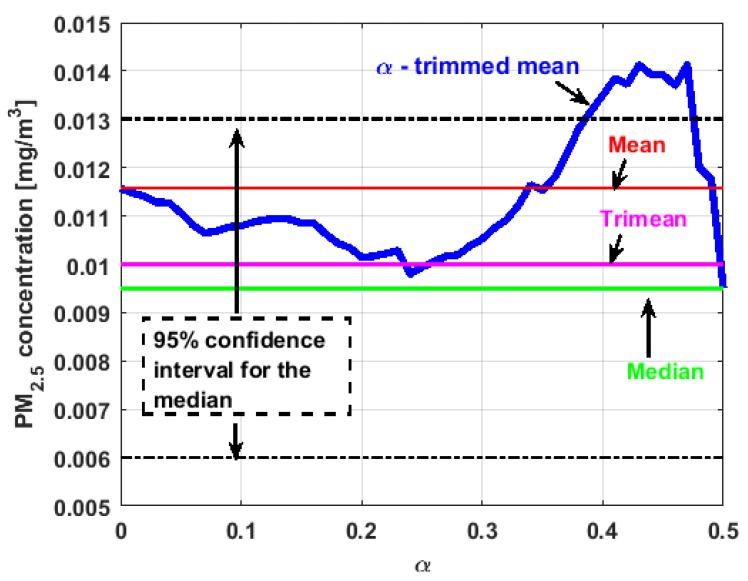
α-trimmed mean of $X_{3}$.

**Figure 20:**
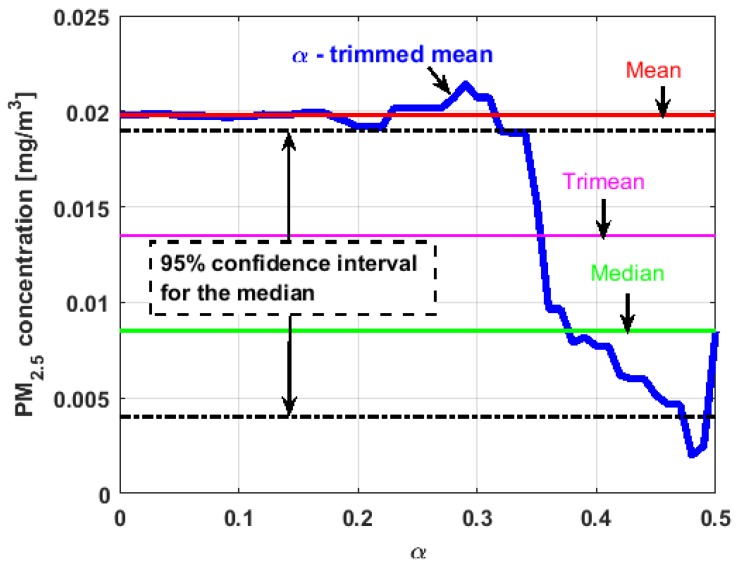
α-trimmed mean of $Y_{1}$.

**Figure 21:**
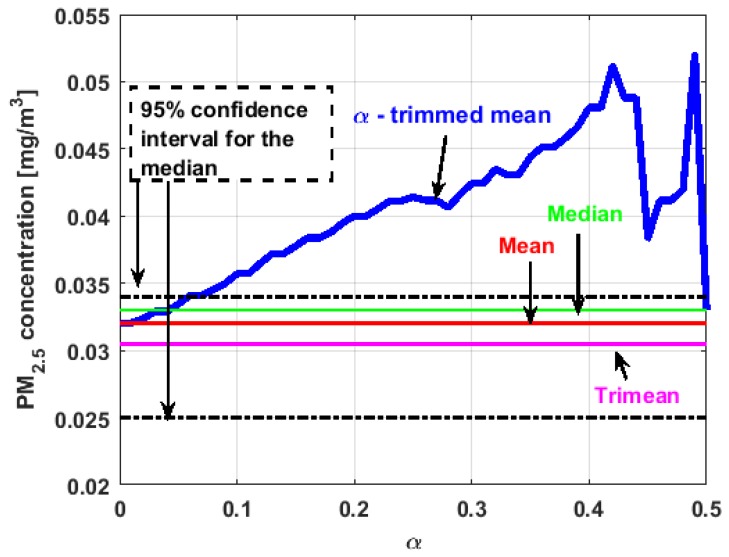
α-trimmed mean of $Y_{2}$.

**Figure 22:**
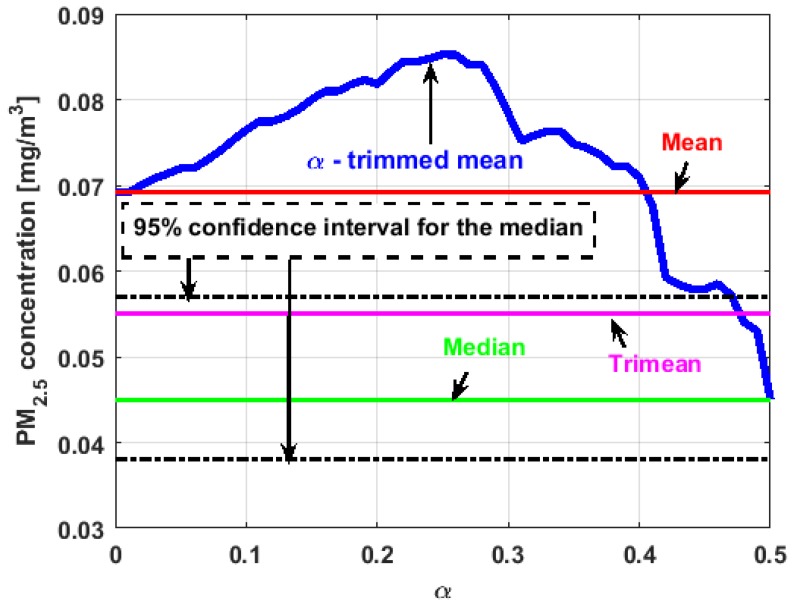
α-trimmed mean of $Y_{3}$.

Figure 17, Figure 18, Figure 19, Figure 20, Figure 21 and Figure 22 show the α-trimmed mean of the variables, along with the mean, median, trimean, and 95% nonparametric confidence interval for the median.

In the graphs shown in Figure 17, Figure 18, Figure 19, Figure 20, Figure 21 and Figure 22, the values of the α-trimmed mean function with low abscissa correspond to the extreme data of the distribution of values of the variable under study, and the values of the α-trimmed mean function with high abscissa correspond to the data close to the center of the distribution.

From Figure 17, Figure 18, Figure 19, Figure 20, Figure 21 and Figure 22, it can be concluded that all the medians and trimeans are within the confidence band. Furthermore, the means of $X_{2}$, $X_{3}$, and $Y_{2}$ are within the confidence band. All this indicates stability of these variables. In contrast, the means of $X_{1}$ and $Y_{3}$ are well above the upper limit of the confidence band, which suggests the greater variability of these variables.

In addition, the most influential observations for the value of the mean of $X_{1}$ are those with the highest pollution values, since the α-trimmed mean function in abscissa low values is decreasing, and by the center of the distribution, the values fall outside the confidence band. This shows again the great variability of $X_{1}$, which has already been observed through other previous arguments.

In addition, $X_{2}$ is the variable that has the most regular behavior of the location measurements. The observations that most influence the value of the median are the lowest, as the function for low abscissa values is increasing. Values near the center have many fluctuations, but are much smaller than those observed in $X_{1}$.

Moreover, $X_{3}$ has many observations with very low values, which leads to the fact that the values of the α-trimmed mean function are far from the lower limit of the confidence band. Similar to $X_{2}$, the function values close to the center of the distribution data suffer from fluctuations, and these fluctuations are similar to those of $X_{2}$ and lower than the fluctuations of the rest of the variables. The difference between $X_{2}$ and $X_{3}$ is that the air pollution values of $X_{2}$, for the most part, are higher than those of $X_{3}$. This fact and the high variability of $X_{1}$ were already anticipated in Figure 4a.

When evaluating the α-trimmed mean function in $Y_{1}$, it is observed that in the central values of the function, it occurs the same as in the variables already analyzed. Specifically, it seems that the most decisive observations for the value of the median are grouped near it. At the extremes, it behaves symmetrically, but through the center of the data, there is a lower level of pollution. All this again suggests high variability.

In addition, $Y_{2}$ has many observations with low values. Thus, the values of the α-trimmed mean function are far from the lower limit of the confidence band. When suppressing extreme values, the function evaluated in low abscissa is increasing, remarking the presence of many observations of values well below the median. It is the only variable in which the median is greater than the mean.

Furthermore, $Y_{3}$ is the variable for which the α-trimmed mean function has the highest range. The initial growth indicates a suppression of low values, which is corroborated by the fact that the function moves away from the lower limit of the confidence band. When for obtaining the α-trimmed mean function, it started to delete values greater than 60% of the extreme data, and this function decreased. This suggests that the central values of the distribution are greater than the median. What has been said here can also be seen in Figure 4b.

In [4], in order to achieve robust linear regression, the *M*-estimation method was used to reduce the influence of outliers in least squares fitting. According to [4], the *M*-estimation method was given in the form of a weight function of residuals, and its performance was satisfactory provided that the distribution of the response was normal and had no outliers. For the case under study in [4], the best model was the robust linear regression using the Talwar *M*-estimator.

However, in this research article, *L*-location estimators were used to estimate the central tendency of the data in a robust manner. Here, it was not assumed that the data fits a normal distribution. In fact, the skewness and kurtosis values shown in Table 1 show that the data do not fit a normal distribution. In addition, the box plots shown in Figure 5 show that some variables have many outliers. What have been explained above is a characteristic of heavy-tail distributions [40]. This situation justified the need to use robust estimators in this article.

Despite the fact that the research objectives in [4] and in this article were different, the use of *M*-estimators and *L*-location estimators gave satisfactory results. In addition, these results demonstrate that these estimators can be applied in cases where it is required to robustly estimate the concentration of PM_2.5_ and the sample size is small, as is the case study in this article.

### 4.4. Robust Analysis of Scale Parameters

Taking into account the obtained results, it was necessary to find an estimate of the dispersion of the variables under study. Although $X_{2}$ and $X_{3}$ could be left out of this analysis, because they do not have enough extreme observations, the estimation of the dispersion of all the variables was carried out.

The measure that is commonly used to describe the variability of a sample of size n of a random variable, Ψ, is the sample standard deviation, given by Equation (3):

(3) $$S_{\Psi }={\left(\frac{1}{n-1}\overset{n}{\underset{i=1}{\sum }}{\left(\psi_{i}-\bar{\psi }\right)}^{2}\right)}^{\frac{1}{2}}$$

$S_{\Psi }$ satisfies both the *shift invariance* condition, $S_{\Psi +\lambda }=S_{\Psi }\;\forall \;\lambda \in \mathbb{R}$, and the *scale equivariance* condition, $S_{\lambda \Psi }=\left|\lambda \right|S_{\Psi }\;\forall \;\lambda \in \mathbb{R}$. According to [40], any statistic satisfying these two conditions is a *dispersion estimate*. The scale estimators used in this subsection were the following [39]:

1. Mean Absolute Deviation:
  (4) $$MAD_{mean}=\frac{1}{n}\sum \left|\Psi_{i}-\bar{\Psi }\right|$$
2. Standard Deviation (see (3)).
3. Median Absolute Deviation:
  (5) $$MAD=median\left\{\left|\Psi_{1}-M_{e}\right|,\ldots ,\left|\Psi_{n}-M_{e}\right|\right\}$$
  where $M_{e}$ is the median of the data.
4. Semi Interquartile Range:
  (6) $$SIR=\frac{Q_{3}-Q_{1}}{2}$$
  where $Q_{1}$ is the first quartile and $Q_{3}$ is the third quartile.
5. Biweight midvariance:
  (7) $$S_{bi}\left(c\right)=\frac{\sqrt{n\sum_{i=1}^{n}{\left(\psi_{i}-MAD\right)}^{2}{\left(1-u_{i}^{2}\right)}^{4}}}{\left|\sum_{i=1}^{n}\left(1-u_{i}^{2}\right)\left(1-u_{i}^{2}\right)\right|}$$
  where:
  (8) $$u_{i}=\frac{\Psi_{i}-MAD}{c\cdot MAD}$$

The family of scale estimators $S_{bi}\left(c\right)$ is based on an *M*-estimator and, according to [42], the scale measurement performed with this estimator has greater efficiency than conventional scale measurements in a wide type of distributions.

Taking into account that approximately the expected value of the MAD statistic is $\frac{2}{3}\sigma$, where σ is the standard deviation of the population, if in Equation (8) it is chosen that $c=k^{2}$, then in order to find the desired estimate, the observations that are at a distance of MAD in more than $\frac{2}{3}k^{2}$ will not be considered. So, if c=9, then only those observations whose distance from MAD is less than 6 times the standard deviation will be considered. As c increases, the values of $u_{i}$ decrease in absolute value, and the value of the denominator of $S_{bi}\left(c\right)$ increases. Therefore, this function decreases.

6. Estimators based on a subrange [57]:
  (9) $$C_{n}^{\alpha }=\frac{1}{\Phi^{-1}\left(0.75\right)-\Phi^{-1}\left(0.75-\alpha \right)}{\left|\Psi_{\left(i+\left[\alpha \cdot n\right]+1\right)}-\Psi_{\left(i\right)}\right|}_{\left(\left[\frac{n}{2}\right]-\left[\alpha \cdot n\right]\right)}$$
  where 0≤α<0, n is the length of the dataset that we want to estimate its spread, $\Psi_{\left(1\right)}\leq \Psi_{\left(2\right)}\leq \ldots \leq \Psi_{\left(n\right)}$ are order statistics [56], […] denotes the integer part, and $\Phi^{-1}\left(p\right)$ is the inverse of standard normal cumulative distribution function, evaluated at the probability values in p.

As α grows, the subtractions between the order statistics shown in Equation (9) are carried out between order statistics that are increasingly separated from each other. Therefore, the observations that are more toward the center of the dataset will be located close to the minimum of the subtractions between the order statistics mentioned above, and also the value of the denominator of Equation (9) will increase. Due to this, the $C_{n}^{\alpha }$ function has less influence from extreme observations as α grows.

In accordance with [57], for α≈0.5 the least median squares (LMS) estimator is obtained. In this paper, the LMS estimator given by Equation (10) was used:

(10) $$LMS\left(\Psi \right)=\frac{1}{2}\underset{i=1,\ldots ,\left[\frac{n}{2}\right]}{\min}\left|\Psi_{\left(i+\left[\frac{n}{2}\right]\right)}-\Psi_{\left(i\right)}\right|$$

This estimator has an expression that is analogous to $C_{n}^{\left[\frac{n}{2}\right]}$, except only for the quotient [57].

Table 4 shows the value of the statistics obtained for each variable. Figure 23, Figure 24, Figure 25, Figure 26, Figure 27 and Figure 28 show estimates of the estimator $S_{bi}$ for all variables, 0<c<18. Figure 29, Figure 30, Figure 31, Figure 32, Figure 33 and Figure 34 show estimates of the estimator $C_{n}^{\alpha }$ for all variables, 0<α<0.5.

Taking into consideration what is shown in the graphs of Figure 23, Figure 24, Figure 25, Figure 26, Figure 27, Figure 28, Figure 29, Figure 30, Figure 31, Figure 32, Figure 33 and Figure 34 and in Table 4, it can be said that for each variable, the estimate $S_{\Psi }$ is greater than the estimate $MAD_{mean}$, and in turn, $MAD_{mean}$ is greater than the other three estimates, noting that the estimates MAD, SIR, and LMS are more robust than the first two.

In addition, $X_{1}$ has a great value of $S_{\Psi }$, which is much greater than the $MAD_{mean}$ estimate, and the latter is, in turn, approximately four times greater than the other three estimates: MAD, SIR, and LMS. Taking into account that these last three estimators are the most robust among all those considered, it can be said that the extreme observations that have, in general, very high values and others very close to zero are very influential in the estimates that include them. The graphs that represent the functions $S_{bi}$ and $C_{n}^{\alpha }$ are very similar, when they are compared to each other for low abscissa values. Therefore, estimates consider most of the observations. In addition, both functions show fluctuations, but then the two families of estimates stabilize around values between MAD and $MAD_{mean}$.

With respect to $X_{2}$, the first thing that can be said is that the point estimates of scale MAD, $MAD_{mean}$, SIR, and LMS are very similar to each other. The graph of the function $S_{bi}$ has two very pronounced maximums for low values of c, while the graph of function $C_{n}^{\alpha }$ also has a maximum for low values of α that is much lower than the two above-mentioned maximums. Moreover, it can be seen how both functions immediately stabilize around the point estimates. The foregoing indicates that there are not many extreme values that influence scale estimates, and that all the scale estimates found are acceptable. Furthermore, the scale estimates of $X_{1}$ that are more robust (i.e., MAD, SIR, and LMS) are slightly greater than the respective scale estimates of the variable $X_{2}$.

With respect to $X_{3}$, it can be said that the value of the robust estimates (i.e., MAD, SIR, and LMS) of this variable are greater than more of the half of the value of the non-robust estimates (i.e., $MAD_{mean}$ and $S_{\Psi }$). The graphs of $S_{bi}$ and $C_{n}^{\alpha }$ have characteristics analogous to the respective graphs of $X_{2}$. It can be seen that $S_{bi}$ has a very pronounced maximum for low values of c, and that and $C_{n}^{\alpha }$ also has a low pronounced maximum for low values of α. In addition, both functions immediately begin to fluctuate around the found point estimates. Therefore, it can be said that there are few extreme values, with high values, that influence the estimates remarkably. However, unlike what happens in the case of $X_{2}$, the estimates obtained with the families of estimators are below MAD, for the case of $C_{n}^{\alpha }$, and below $MAD_{mean}$, for the case of $S_{bi}$. Therefore, the estimators $S_{bi}$ and $C_{n}^{\alpha }$ are not so similar. Moreover, the most robust scale estimates are similar to each other in the case of $X_{1}$ and $X_{3}$, but these robust estimates have somewhat greater values than those corresponding to the case of $X_{2}$.

In addition, the point estimates of scale of $Y_{1}$ show appreciable differences. It can be seen that $S_{\Psi }$ is appreciably higher than the rest of the estimators. In addition, the $MAD_{mean}$ and SIR estimates are similar, the MAD value is half the value of the previous ones, and the LMS value is half the MAD value. All this indicates that there are influential observations in the scale estimates. The graphs of $S_{bi}$ and $C_{n}^{\alpha }$ are similar to the respective graphs of $X_{3}$, even in the difference with $X_{2}$. Therefore, there are a few extreme high observations that produce estimates above the possible real value. The $S_{bi}$ family oscillates around $MAD_{mean}$ and the $C_{n}^{\alpha }$ family oscillates around MAD.

Moreover, $Y_{2}$ has quite different point estimates of scale, although the most robust estimators (i.e., MAD, SIR, and LMS) are very similar to each other, and slightly greater than the point estimators of scale of the variables that have been previously analyzed. The graphs of the functions $S_{bi}$ and $C_{n}^{\alpha }$ are different from the other graphs of the rest of the variables. It can be seen that $C_{n}^{\alpha }$ is the graph that is most influenced by extreme values and the high values of the variable. By suppressing the extreme observations, the $C_{n}^{\alpha }$ estimates are around $MAD_{mean}$. The point scale estimates seem somewhat greater than those of the variables previously analyzed, but lower than those of the variable $Y_{3}$.

Furthermore, by removing the $S_{\Psi }$ scale estimate of variable $X_{1}$, the point scale estimates of $Y_{3}$ are greater than all the remaining ones. In addition, if only the most robust estimates (i.e., MAD, SIR, and LMS) are considered, then it can be seen that the differences with the estimates of the rest of the variables are 50% higher than the value of the estimates that so far were the highest; that is, those of the variable $Y_{2}$. In addition, it can be said that $Y_{3}$ has similar characteristics to other variables. For example, there were observations with extremely high values that greatly influenced the estimates that took them into account. In addition, the $S_{bi}$ family tends to be bounded by point estimates of scale and to oscillate around $MAD_{mean}$. Moreover, the $C_{n}^{\alpha }$ family tends to have oscillations, and it tends to MAD.

Before moving on to the next section, it is important to highlight that, taking into account the previous comments, the variables under study can be classified according to their scale of variation. In this sense, $X_{2}$ is classified as the smallest. Second, the variables that have the least variation, but with greater variation than $X_{2}$, are $X_{1}$ and $X_{3}$. $Y_{1}$ and $Y_{2}$ can be placed in third and fourth place, respectively. Finally, the variable with the greatest variation is $Y_{3}$, which has both the greatest variation and most of the points of influence. The results of this paper are compatible with those of [37].

## 5. Conclusions

In this article, data from PM_2.5_ concentration measurements performed in La Carolina Park, Quito, Ecuador, were analyzed using robust statistics techniques. First, a statistical summary of the data was shown. In addition, it was found that the distributions of the data were not normal and that they could be heavy-tailed distributions.

In a preliminary analysis of the data, it was seen that all the extreme observations of the variables under study corresponded to high values of air pollution by PM_2.5_ concentrations. In addition, it was seen that $X_{1}$ was the only variable that had extreme values for low air pollution levels. Furthermore, the lack of normality was a characteristic that was common of all the variables.

From hypothesis tests and the establishment of nonparametric confidence intervals, it was concluded that the median of $Y_{3}$ was the greatest. It was also concluded that the median of $Y_{2}$ was different from the other medians, and that the medians of $X_{1}$ and $X_{2}$ are equal to each other but different from all other medians. In addition, it was shown that the medians of $X_{3}$ and $Y_{1}$ are equal to each other and smaller than the other medians.

From the analysis carried out in this article, it was observed that the numerical values of $X_{1}$ were the only ones that sometimes exceeded all air quality limits established by the Quito Air Quality Index (QAQI). All the numerical values of $X_{2}$ except one of them were below the Acceptable level, and the numerical values of $X_{3}$ rarely exceeded the Desirable level. Few numerical values of $Y_{1}$ exceeded the Acceptable level, and many of its observations were at the Desirable level. Most of the observations of $Y_{2}$ were below the Caution level, and the vast majority were below the Acceptable level. The variable with the highest observations after $Y_{1}$ was $Y_{3}$, although its observations did not exceed the Alarm level. Furthermore, a Wilcoxon rank-sum test showed that air pollution due to $X_{1},$ $X_{2}$, and $X_{3}$ was equal to air pollution due to $Y_{1},$ $Y_{2}$, and $Y_{3}$, at the α=0.05 significance level.

In this paper, it was shown that neither the streets that border the park nor the park itself (i.e., $X_{1},$ $X_{2}$, $X_{3}$, $Y_{1},$ $Y_{2}$, and $Y_{3}$) were on Alert level. According to categories established by QAQI, the most critical case was $Y_{3}$ ($Y_{3}$ refers to a route along which measurements were performed in the street Avenida Naciones Unidas), which was at the Caution level. Therefore, measures that help to improve air quality in the region where La Carolina Park is located must be taken.

Once this first analysis was completed, it was decided to provide some robustness with respect to the estimates of both the central tendency of the data and its dispersion, because it is very important to give estimates that are practically immune at least to small variations in the data. Thus, unless a natural disaster occurs, such as an earthquake or a volcanic eruption, among others, regardless of the possible distribution of the data, estimates of the central tendency of the data and its dispersion will be limited by intervals that guarantee, with great certainty, that the true values of the magnitudes of the quantities under study are not very far from their estimates.

For centralization measures, the family of α-trimmed means and point estimates of the mean, median, and trimean were considered. For scale estimations, five point estimators were considered, which are not all robust. These estimators were the following: standard deviation, mean absolute deviation, median absolute deviation, semi interquartile range, and least median squares. Moreover, two families of robust estimators were considered: biweight midvariance estimators and estimators based on a subrange.

The results of this research showed that robust estimates of location of $X_{1}$ were very dependent on the number of observations that were not considered, although all these estimates were between the Desirable level and Acceptable level. On the other hand, robust estimates of the scale of $X_{1}$ were around 0.006 units. These data indicated the possibility that the distribution of $X_{1}$ is a distribution of heavy tails. This argument was corroborated by the high values of its skewness and kurtosis.

With respect to $X_{2}$, most of the location estimates were in the nonparametric confidence band established for that variable. Since $X_{2}$ had barely extreme observations, the values of the robust location and scale estimators of this variable were all similar. In this case, all the robust location estimations were at the Acceptable level, and the robust scale estimations were close to $0.003\;\mathrm{mg}/m^{3}$. $X_{1}$ and $X_{2}$ would have their location measurements at the Acceptable level; the scale of $X_{2}$ is half that of the scale for $X_{1}$, and their medians cannot be medians of the other variables that have been analyzed.

The above agrees with the fact that the difference between $X_{1}$ and $X_{2}$ is the street Avenida de los Shyris, where $X_{2}$ refers to a route that is right next to La Carolina Park and $X_{1}$ refers to a route where there is a highly commercial area. Therefore, the measurement results of $X_{1}$ are greater than the measurements results of $X_{2}$, causing the displacement of the frequency distribution of $X_{1}$ to a higher value zone, and that the observations of $X_{1}$ with low numerical values appear as lower extreme data. Moreover, the above entails the appearance of a greater variability in the $X_{1}$ data compared to the $X_{2}$ data. This explanation demonstrates that the park is working as an air pollution filter.

Most of the robust location estimates of $X_{3}$ and $Y_{1}$ were in the confidence band established by nonparametric statistics, and all estimates were at the Desirable level. Therefore, the medians of the other variables cannot be medians of these two variables. In $Y_{1}$, there seemed to be a breaking point from which robust location estimates were near the mean on the left, and near the median on the right. In addition, the scale estimates of $Y_{1}$ were somewhat higher than the scale estimates of $X_{3}$. This happened because the range of observations of $Y_{1}$ for high pollution values was longer on the right than the range of $X_{3}$. These two variables also showed a similarity regarding the geographical area, because the observations of both variables were taken on wooded areas of the park.

With respect to robust scale estimates of $X_{3}$, it can be said that these were similar to the robust scale estimates of $X_{1}$. In addition, robust scale estimates of $Y_{1}$ were significantly greater than robust scale estimates of $X_{3}$. $X_{3}$ and $Y_{1}$ were the variables with the best air quality levels.

Furthermore, the robust location estimates of $Y_{2}$ that came from the α-trimmed means were above the nonparametric confidence interval, indicating that there were few high observations and many low observations. However, almost all the estimates were below the Acceptable level. $Y_{2}$ showed analogies with $Y_{1}$ in terms of the distribution of the data, but with a shift of them toward higher values. In spite of the fact that the measurements of $Y_{2}$ were performed in the middle of the park, in the area where these measurements were performed, there was not any wooded region. Once again, it is observed that where there are more trees and vegetation in an area, the lower the air pollution level in that area.

The robust scale estimates of $Y_{2}$ were similar to each other and larger than those of the other variables, except for $Y_{3}$.

In addition, $Y_{3}$ showed robust location estimates from the α-trimmed means that were above the nonparametric confidence interval. Moreover, $Y_{3}$ is the variable that produced location estimates significantly greater than those of the other variables, being able to fall between the Acceptable level and the Caution level. This variable was the one with the greatest values of scale estimates.

The above is in accordance with the geographical situation in which the street Avenida Naciones Unidas is located and the route to which $Y_{3}$ refers. The street Avenida Naciones Unidas goes from east to west in the city, and it is well known that in Quito, the circulation speed of cars, buses, and trucks in that direction is slower than in any other direction. Therefore, there are more traffic jams when driving in that direction and, as a result, citizens on that street are much more exposed to air pollution.

At this point, it is important to say that in this article, robust confidence intervals were not included. Therefore, this is a task that remains pending for future research works. However, these confidence intervals are based on robust location and scale estimators, and these robust estimators in turn depend on the order statistics that were used to find the nonparametric confidence intervals. Therefore, it seems that there must be a close relationship between both kinds of confidence intervals.

The results of this study confirm that the efficiency of all urban dynamics (that is, mobility, recreation, cultural activities, and work activity, among others) must be subject to appropriate environmental quality standards. In the case of La Carolina Park, the quality and diversity of the activities that can be carried out in the park, together with the urban offer of the sector in which this part is located (which offers great competitiveness in terms of the following activities: commercial, labor, hotel, banking, governmental, and educational, among others), make this urban park one of the most visited spaces in Quito. Nevertheless, two critical areas were identified in the study:

1. First critical area: For the case of La Carolina Park, the area near the intersection between Avenida Naciones Unidas and Avenida de los Shyris. Various sports are practiced in this area, which represents activities that demand more oxygen. This part of the park contains less woodland that mitigates the pollution that is generated in these two avenues.
2. Second critical area: The paths of the roads that surround the park (most of them very wide), where from Monday to Friday they are very busy by people who work and/or live in the area. In these paths, the access to the premises located on the ground floor and the bus stops (specifically in Avenida Naciones Unidas, in Avenida de los Shyris, and in the northern part of the Avenida Rio Amazonas) are very exposed to the air pollution that is generated in these paths.

Therefore, as a result of the study carried out, it is recommended that these two areas have a new design and environmental management to reduce pollution emitting agents, and to mitigate and/or reduce the exposure of people to polluted air. As the reduction of pollution sources is subject to the urban structure of the city of Quito (which is a long, narrow city), and as there is also a need for more efficient and less polluting public transport, it is suggested that the urban decision makers, owners, and citizens in general take control (C) and mitigation (M) measures, such as:

- (C1) Urban decision makers: Control the entry of polluted air to the food preparation and sale premises that are located on the ground floor by regulating the design of the accesses, especially when the access is direct from a route large vehicular flow.
- (C2) Owners (especially of food premises on ground floors): Control food processing processes so that they are located in areas less exposed to polluted air.
- (C3) Owners (of premises and/or homes): Consider natural ventilation habits (preferably cross-ventilation) of the buildings, taking into account the hours when pollution levels are lower (from 5:00 to 6:00 and from 20:00 to 21:00).
- (M1) Urban decision makers: Mitigate exposure to pollution in the northeast part of the park, redesigning the placement of trees with more foliage at the edge of the park and promoting that sports activities be protected from pollution by a green filter. To achieve this goal, it is important that urban design can redirect activities that take the edge of the park as a unit of measurement, such as: walking, running and/or walking domestic animals.
- (M2) Urban decision makers: Mitigate exposure to pollution through elements that protect people from polluted air, especially in the busiest sidewalks, which are on the urban edge of the roads surrounding the park.
- (M3) Urban decision makers: Mitigate exposure to pollution by informing the population about the levels of pollution in each area of the park. In addition, do this taking into account the precautions that must be taken into account to develop urban dynamics in the best possible environmental conditions.

## Figures and Tables

**Figure 23:**
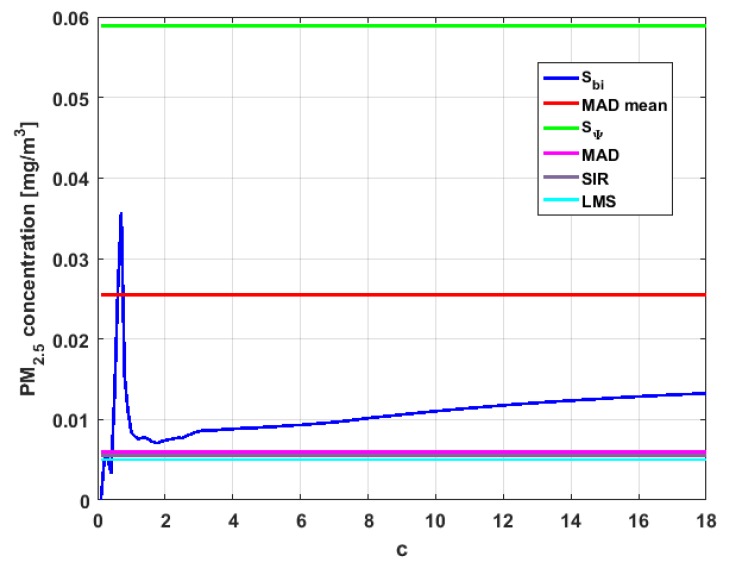
$S_{bi}$ estimates of $X_{1}$.

**Figure 24:**
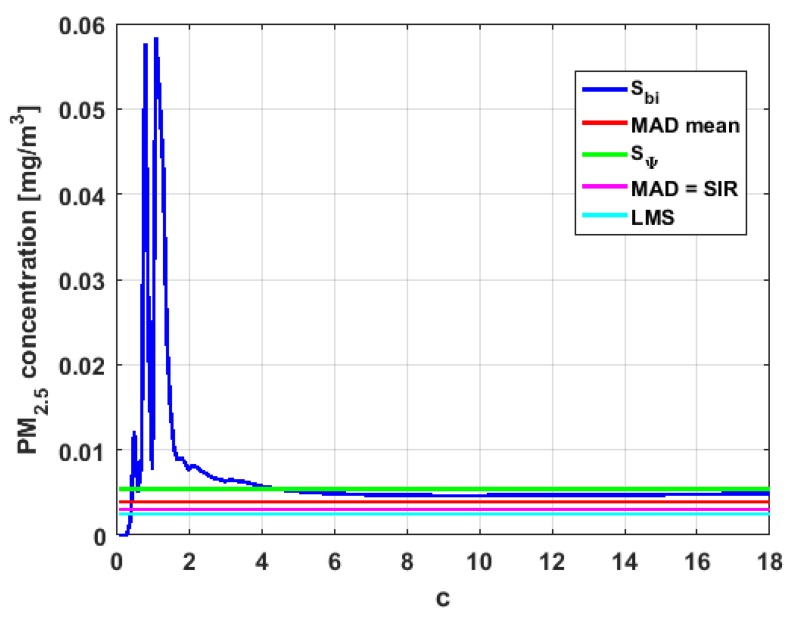
$S_{bi}$ estimates of $X_{2}$.

**Figure 25:**
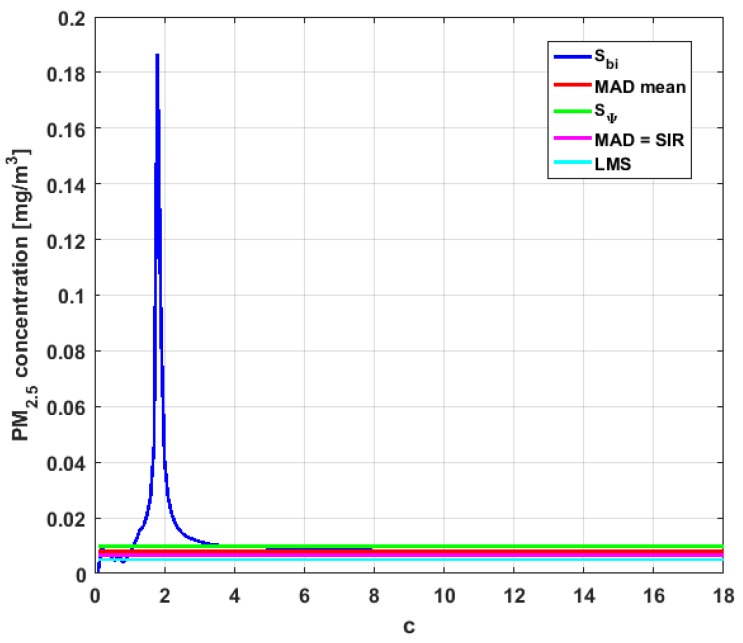
$S_{bi}$ estimates of $X_{3}$.

**Figure 26:**
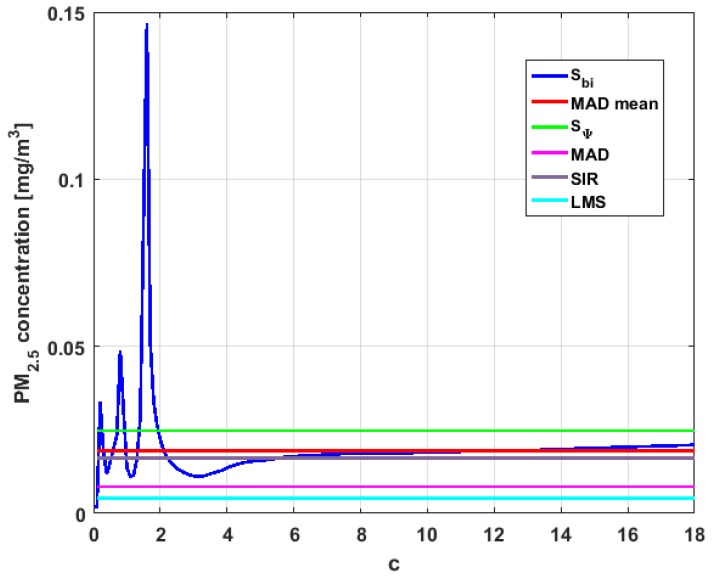
$S_{bi}$ estimates of $Y_{1}$.

**Figure 27:**
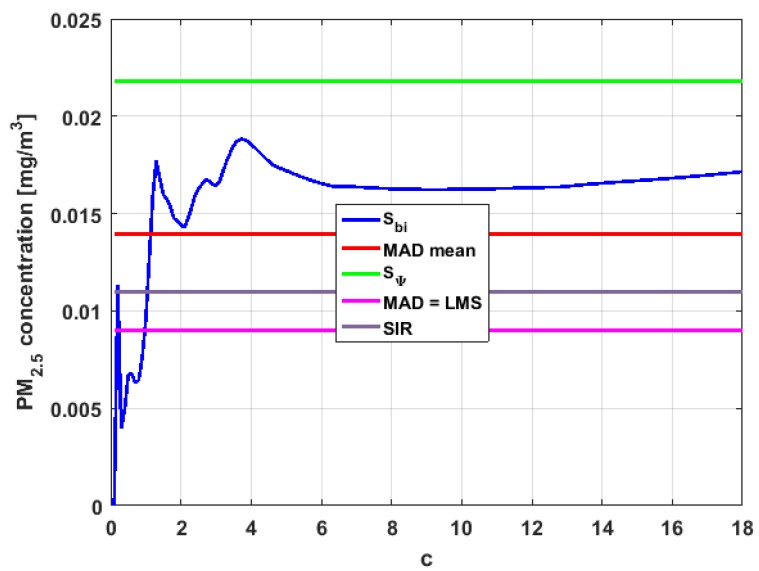
$S_{bi}$ estimates of $Y_{2}$.

**Figure 28:**
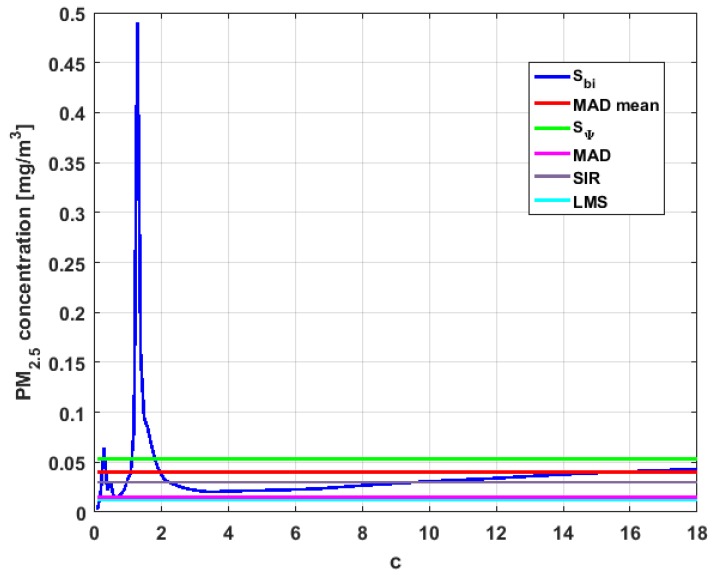
$S_{bi}$ estimates of $Y_{3}$.

**Figure 29:**
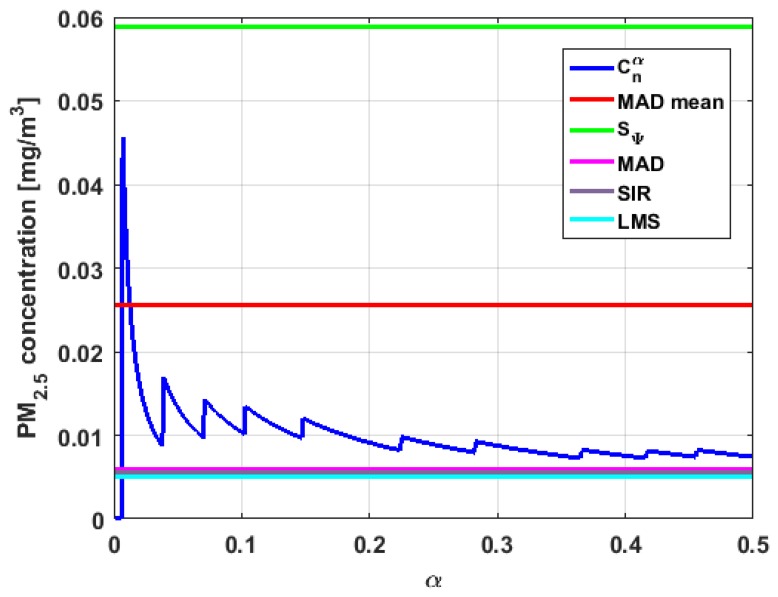
$C_{n}^{\alpha }$ estimates of $X_{1}$.

**Figure 30:**
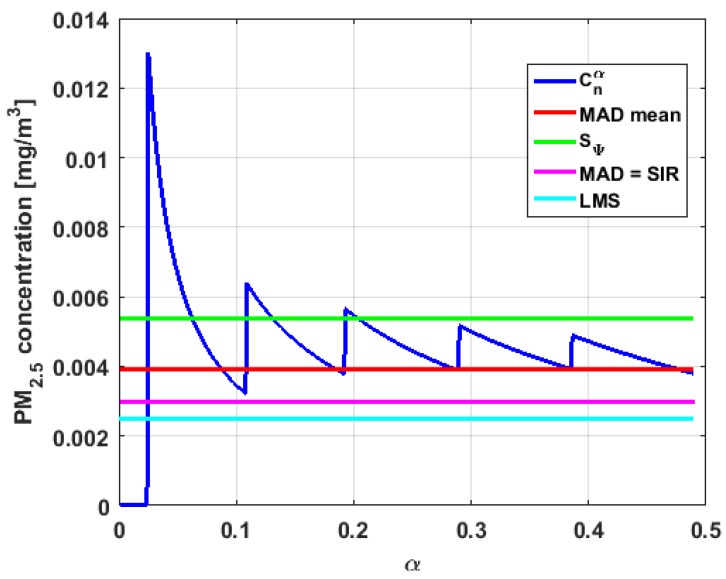
$C_{n}^{\alpha }$ estimates of $X_{2}$.

**Figure 31:**
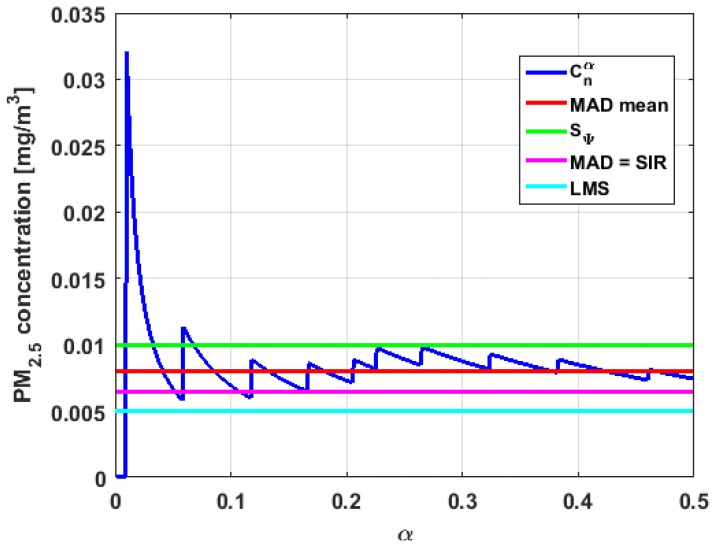
$C_{n}^{\alpha }$ estimates of $X_{3}$.

**Figure 32:**
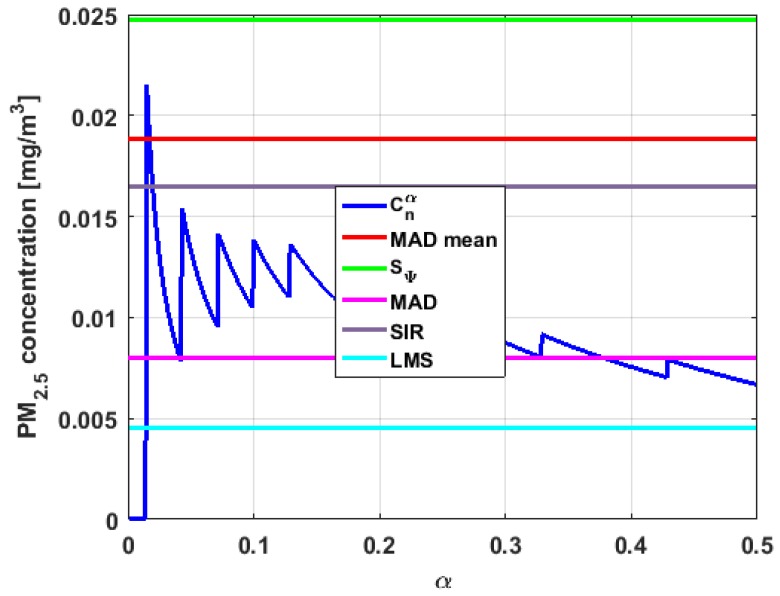
$C_{n}^{\alpha }$ estimates of $Y_{1}$.

**Figure 33:**
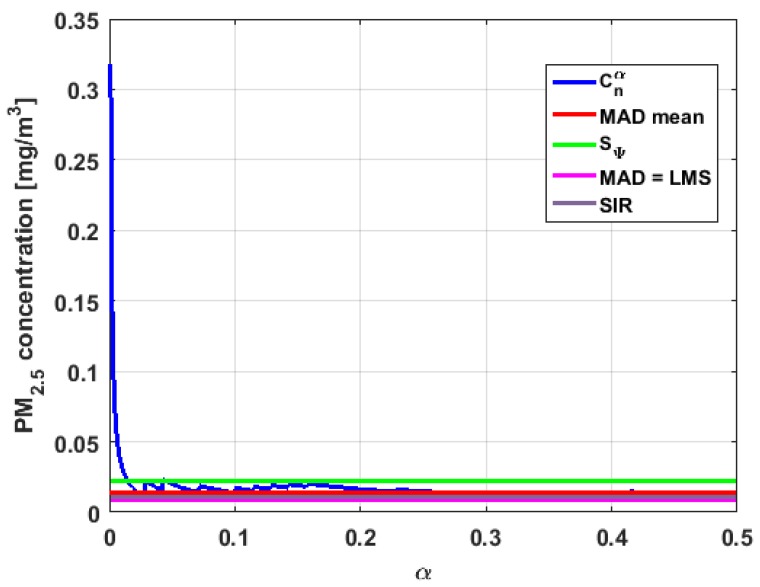
$C_{n}^{\alpha }$ estimates of $Y_{2}$.

**Figure 34:**
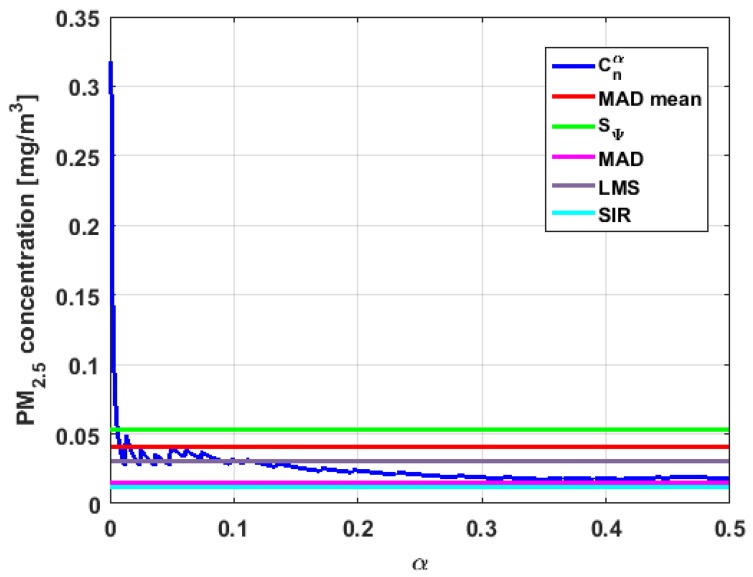
$C_{n}^{\alpha }$ estimates of $Y_{3}$.

**Table 1:** Summary statistics.

Variable	Count	Average	Median	Standard Deviation	Skewness	Kurtosis	Minimum	Maximum
$X_{1}$	156	0.0485	0.0360	0.0589	4.9970	29.0421	0	0.4390
$X_{2}$	83	0.0373	0.0370	0.0054	1.5598	8.8214	0.0280	0.0640
$X_{3}$	102	0.0116	0.0095	0.0099	1.0046	3.3853	0	0.0430
$Y_{1}$	70	0.0198	0.0085	0.0247	1.9513	7.4982	0	0.1190
$Y_{2}$	70	0.0321	0.0330	0.0218	2.4862	13.6409	0.0030	0.1490
$Y_{3}$	82	0.0692	0.0450	0.0531	1.6625	5.0901	0.0200	0.2450
**Total**	607	0.0375	0.0340	0.0432	4.8314	35.3189	0	0.4390

**Table 2:** Rejection limits at the significance level α=5%.

Variable	Lower Rejection Limit	Upper Rejection Limit	Length of the Confidence Interval	*p*-Value
$X_{1}$	0.034	0.037	0.003	0.2979
$X_{2}$	0.036	0.038	0.002	0.1875
$X_{3}$	0.006	0.013	0.007	1.0000
$Y_{1}$	0.004	0.019	0.015	1.0000
$Y_{2}$	0.025	0.034	0.009	0.5504
$Y_{3}$	0.038	0.057	0.019	1.0000

**Table 3:** Air pollution due to groups X and Y.

	Air Pollution Categories by PM_2.5_			
Groups	Desirable Level	Acceptable Level	Caution Level	Total
$Y=\left\{Y_{1},Y_{2},Y_{3}\right\}$	$1\;\left(\left\{Y_{1}\right\}\right)$	$1\;\left(\left\{Y_{2}\right\}\right)$	$1\;\left(\left\{Y_{3}\right\}\right)$	3
$X=\left\{X_{1},X_{2},X_{3}\right\}$	$1\;\left(\left\{X_{3}\right\}\right)$	$2\;\left(\left\{X_{1},X_{2}\right\}\right)$	0 ({∅})	3
**Total**	2	3	1	6
∅ is the empty set.				

**Table 4:** Scale estimates.

Variable	$MAD_{mean}$	$S_{\Psi }$	MAD	SIR	LMS
$X_{1}$	0.0255	0.0589	0.0060	0.0055	0.0050
$X_{2}$	0.0039	0.0054	0.0030	0.0030	0.0025
$X_{3}$	0.0080	0.0099	0.0065	0.0065	0.0050
$Y_{1}$	0.0189	0.0247	0.0080	0.0165	0.0045
$Y_{2}$	0.0139	0.0218	0.0090	0.0110	0.0090
$Y_{3}$	0.0403	0.0531	0.0150	0.0300	0.0120
